# Relationships between heart shape, function, and disease in 38,858 UK biobank participants

**DOI:** 10.1016/j.jocmr.2025.101919

**Published:** 2025-06-02

**Authors:** Richard Burns, Laura Dal Toso, Charlène A. Mauger, Alireza Sojoudi, Avan Suinesiaputra, Steffen E. Petersen, Julia Ramírez, Patricia B. Munroe, Alistair A. Young

**Affiliations:** a School of Biomedical Engineering and Imaging Sciences, King’s College London, London, UK; b William Harvey Research Institute, Faculty of Medicine and Dentistry, Queen Mary University of London, London, UK; c Institute for Biomedical Engineering, University and ETH Zurich, Zurich, Switzerland; d Department of Anatomy and Medical Imaging, University of Auckland, Auckland, New Zealand; e Overjet, Palo Alto, USA; f NIHR Barts Biomedical Research Centre, Queen Mary University London, London, UK; g Barts Heart Centre, St Bartholomew’s Hospital, Barts Health NHS Trust, London, UK; h Aragon Institute of Engineering Research, University of Zaragoza, Zaragoza, Spain; i Centro de Investigación Biomédica en Red, Biomateriales, Bioingeniería y Nanomedicina, Zaragoza, Spain

**Keywords:** Strain, MAPSE, TAPSE, Shape

## Abstract

**Background:**

Cardiac functional metrics such as ejection fraction, strain, and valve excursion are important diagnostic and prognostic measures of cardiac disease. However, they ignore a large amount of systolic shape change information available from modern cardiovascular magnetic resonance (CMR) examinations.

We aimed to automatically quantify multidimensional shape and motion scores from CMR, investigate covariates, and test their discrimination of disease in the UK Biobank compared against standard functional metrics.

**Methods:**

An automated analysis pipeline was used to obtain quality-controlled three-dimensional left and right ventricular shape models in 38,858 UK Biobank participants, 5149 of whom had one or more diagnoses of cardiovascular or cardiometabolic disease. Principal component analysis was used to obtain a statistical shape atlas and quantify each participant’s left and right ventricular shape at both end-diastole and end-systole simultaneously. Systolic strain was obtained from arc length changes computed from the shape model, and mitral/tricuspid annular plane systolic excursion (MAPSE/TAPSE) was computed from the displacement of the valves. Discrimination for prevalent disease was quantified using linear discriminant analysis area under the receiver operating characteristic curve.

**Results:**

The first 25 principal component scores captured >90% of the total shape variance. Significantly stronger discrimination for atrial fibrillation, heart failure, diabetes, ischemic disease, and conduction disorders (p<0.001 for each) was obtained using shape scores compared with volumes, ejection fractions, strains, MAPSE, and TAPSE.

**Conclusion:**

Automatically derived shape and motion z-scores capture more discriminative information on disease effects than standard metrics, including volumes, ejection fraction, strain and valve excursions.

## Graphical abstract

**Figure:**
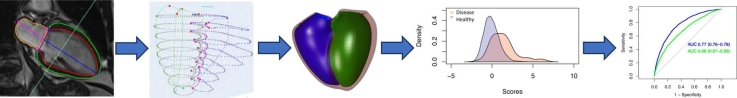

## 1 Introduction

Heart function is commonly quantified by ventricular mass, volume, ejection fraction (EF), myocardial strain, and mitral/tricuspid annular plane systolic excursions (MAPSE/TAPSE) [1]. These metrics provide useful diagnostic and prognostic information on cardiac disease [2], [3]. Strain is commonly computed from image feature tracking or tissue tagging in cardiovascular magnetic resonance (CMR) imaging [1], or speckle tracking in echo [2], and is a more sensitive indicator of adverse events than EF in hypertension [4], hypertrophic cardiomyopathy [5], and cardio-oncology toxicity [6]. However, MAPSE may be more predictive of death and hospitalization for heart failure than strain [7] and may contain additional prognostic information for death in hypertensive patients [8]. The relationships between geometry, EF, strain and motion are complex [9]. Different metrics vary differently with covariates such as afterload and body habitus [10], [11]. It is currently unclear what is the best metric for evaluation of heart disease, and better functional metrics are needed to provide more complete evaluation of cardiac dysfunction [12], [13].

Statistical shape atlases enable quantification of most of the multidimensional heart shape information available in modern medical imaging methods such as CMR [14], [15]. The development of these techniques has been facilitated by large cohort studies such as the Multi‐Ethnic Study of Atherosclerosis (MESA) [16] and the UK Biobank [17], which utilized CMR to study the effects of disease on heart structure and function. Previous studies have shown that atlas shape scores have stronger associations with risk factors such as hypertension, hyperlipidaemia, diabetes, smoking and obesity than standard mass and volume metrics in 1991 participants of MESA [18] and 4329 participants of the UK Biobank [19]. Shape models in ∼1500 volunteers have also shown novel relationships with adiposity and titin-truncating variants [20], [21]. In particular, atlas-based shape scores have shown independent prognostic information for prediction of future adverse events in 4618 MESA participants [22] and in 1021 patients post-acute myocardial infarction [23]. Fully automated analysis pipelines now enable computation of heart shape and scores in >10,000 UK Biobank participants [24], [25], [26]. However, previous studies have not compared shape atlas scores with common functional metrics such as strain and MAPSE for ability to discriminate disease.

The aim of this study was to investigate the sensitivity of atlas metrics of cardiac function to disease, and examine their covariates. We hypothesized that atlas-based shape scores would have stronger discrimination of disease than standard functional metrics of mass, volume, EF, strain and MAPSE/TAPSE. We developed an automated analysis pipeline and generated customized shape models and a statistical shape atlas in 38,858 UK Biobank participants. The atlas captured right and left ventricular shape at both end-diastole and end-systole. Standard functional metrics were computed automatically from the shape models. This enabled direct comparison between atlas scores and standard metrics in linear discriminant analysis evaluation of disease discrimination. We show that atlas scores enable more sensitive discrimination of disease effects on heart shape and function than standard metrics, and show how they can be incorporated into the clinical workflow and used to increase the power of studies to detect disease or treatment effects.

## 2 Methods

**Fig. 1:**
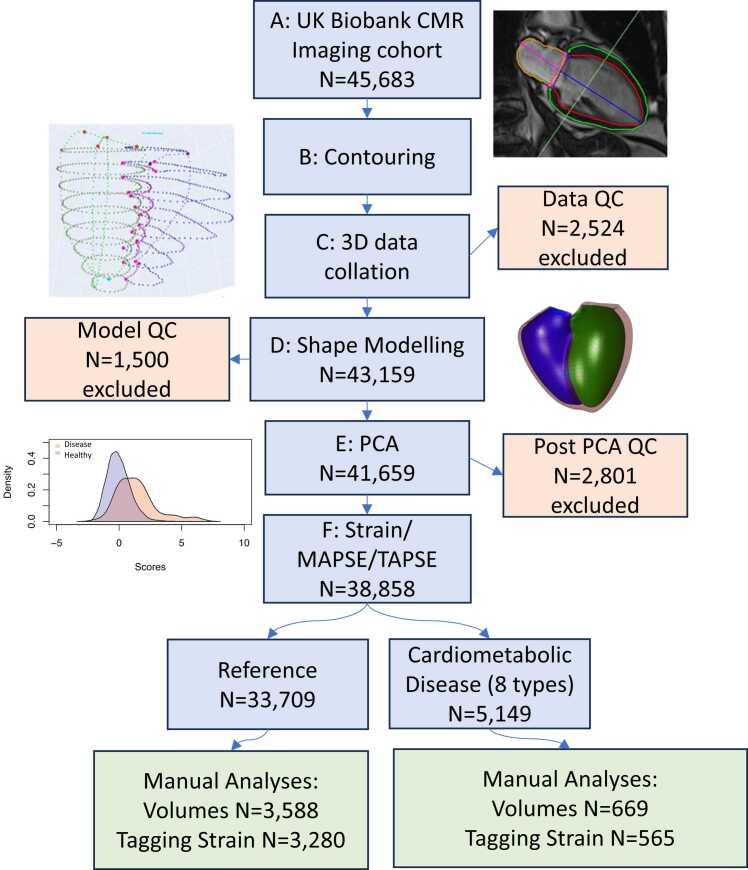
Automated analysis pipeline. *CMR* cardiovascular magnetic resonance, *PCA* principal component analysis, *MAPSE* mitral annular plane systolic excursion, *TAPSE* tricuspid annular plane systolic excursion, *PCA* principal component analysis, *IQR* interquartile range. *QC* quality control. Image reproduced by kind permission of UK Biobank ©.

An overview of the automated analysis pipeline is shown in Fig. 1. The pipeline steps are detailed below. Briefly, CMR images (Fig. 1A) of UK Biobank participants were automatically analyzed using a previously validated [27] deep learning convolutional neural network algorithm (cvi42 Version 5.11 1505) (Fig. 1B). Contours of the right and left ventricles and valve landmarks were extracted from short and long axis images and merged in 3D (Fig. 1C). A biventricular shape model was customized (Fig. 1D) to each case using diffeomorphic registration. Principal component analysis (PCA) was used on the ∼5800 resulting model vertices (Fig. 1D) for unsupervised dimension reduction resulting in a set of PC shape scores for each participant. Participants with cardiovascular or cardiometabolic disease endpoints (eight types), as well as participants with no reported cardiovascular or cardiometabolic disease (reference) were used for subsequent linear discriminant analysis for each disease type.

### 2.1 Study population

This post hoc cross-sectional study evaluated data from the UK Biobank (application 2964); a large-scale prospective cohort study with 500,000 participants aged 40–69 at time of enrollment with detailed health, lifestyle, physical measures, and biological samples. The study design and data collection methods have been described previously [28], [29]. At the time of analysis, 45,683 individuals had available CMR images (Fig. 1A) [29]. All participants gave written informed consent and the appropriate institutional review boards approved the study protocol (National Research Ethics Service North-West 11/NW/0382). Systolic blood pressure was averaged between manual and automated readings taken at the imaging visit and adjusted by adding 15 mmHg if the participant was taking blood pressure altering medication.

Cardiovascular or cardiometabolic disease prevalence was determined for eight categories as follows: atrial fibrillation; heart failure; a composite of myocardial infarction and/or ischemic heart disease; hypertrophic cardiomyopathy; dilated cardiomyopathy; type 2 diabetes mellitus; conduction disease comprising significant atrioventricular block, bundle branch block and fascicular block; and a ventricular arrhythmia composite including ventricular arrhythmia, implanted cardiac defibrillator, sudden cardiac death and/or cardiac arrest. These were chosen to represent common disease categories of interest known to have pathophysiological effects on heart shape and function. Disease categories were determined using ICD9 & ICD10 codes from hospital episode statistics data from the National Health Service (NHS) through the UK Biobank. A reference cohort was also identified with no reported cardiovascular or cardiometabolic disease (the reference sub-cohort).

### 2.2 CMR imaging and automated image analysis

The UK Biobank CMR protocol has been described previously [29]. Briefly, all imaging was performed on a wide bore 1.5T scanner (MAGNETOM Aera, Syngo Platform VD13A, Siemens Healthineers, Erlangen, Germany) using a phased-array cardiac coil. Retrospectively gated balanced steady state free precession cine images were acquired with three long axis orientations (horizontal long axis, vertical long axis, LV outflow tract) and a short axis stack covering both the RV and the LV (6 mm thickness for long axis, 8 mm thickness for short axis, flip angle 80°, TR/TE = 2.6/1.1ms, temporal resolution 32 ms interpolated to 50 phases per cardiac cycle, pixel size 1.8 × 1.8 mm). Each slice was acquired in a separate breath-hold.

Contours and landmarks were automatically identified using cvi42 post-processing software (Version 5.11 1505, Circle Cardiovascular Imaging Inc., Calgary, Canada). This software used deep learning convolutional neural network algorithms for fully automated analysis, which has been previously validated [27]. Contours were identified in short and long axis (two-chamber, three-chamber and four-chamber) images. Tricuspid and aortic valve points were defined from landmarks delineated on the two-chamber and three-chamber long axis views respectively, and mitral valve points were defined on all long axis images. A LV endocardial apex point was defined on the four-chamber image. Since RV free wall myocardium was not detected by the machine learning algorithms, the RV free wall epicardial surface was imputed by displacing the RV endocardial points outwards by 3 mm. The cvi42 software provided a report containing ventricular volumes and LV mass computed using slice summation of the short axis contours. The ED and ES frames were selected as the frames with the highest and lowest volumes computed by cvi42.

### 2.3 Automated biventricular shape analysis and PCA

A biventricular shape model consisting of a subdivision surface template mesh was automatically customized to each case as described previously [30]. Briefly, breath-hold misregistration was automatically corrected and the shape model geometry was customized using diffeomorphic least squares minimization of the distances between the shape model and the contour points from all the short axis and long axis slices.

A biventricular statistical shape atlas was constructed using PCA as previously described [19]. ED shapes were co-registered using Procrustes alignment (without scaling), and the aligned ED and ES shape model vertices were concatenated into a single shape vector. Singular value decomposition of the covariance matrix resulted in a relatively small number of PC scores that described the majority of shape variation across all participants, while accounting for correlations between points in the shape model. The first component explained the most of total shape variance, and each subsequent PC successively explained less variance. For each participant, PC scores were calculated which quantified the amount of each PC present in that individual. The PCs which captured >90% of the total variance were kept for functional analysis.

A series of quality control checks were performed at different stages of the pipeline (details provided in Supplementary Material).

### 2.4 Geometric shortening strain

Geometric % shortening strain was calculated as percent change in arc length:

(1) GLS or GCS = ((EDL-ESL)/EDL) × 100%

where GLS and GCS denote global longitudinal and circumferential strains respectively, and EDL and ESL are the corresponding endocardial surface arc lengths at ED and ES respectively. Note % shortening was defined to be positive for contraction. Previous studies have found good agreement between geometric global strain and feature tracking as well as tagging estimates [31], [32].

### 2.5 MAPSE and TAPSE calculation

Mitral/tricuspid annular plane systolic excursion (MAPSE/TAPSE) was calculated as the mean 3D displacement of valve points from ED to ES in mm.

### 2.6 Covariate analysis

Univariate and multivariate linear regressions were performed using R (version 4.2.1) [33] to quantify the strengths of relationships between functional metrics and covariates, including age, sex, height, body mass index, and afterload, in the disease-free reference sub-cohort. Afterload, or myocyte stress at ES, is known to be correlated with many functional metrics. Here, we estimate afterload using the following Arts formula [34]:

(2) $$\mathrm{Afterload}=\mathrm{SBP}\left(1+\frac{3\;(\mathrm{LVESV})}{\mathrm{LVWallVolume}}\right)$$

where SBP represents systolic blood pressure and LV ES volume and LV wall volume (at ED) were estimated from the model using numerical integration. This formula has been shown to produce accurate estimates of myocyte stress at ES in a variety of geometries [34], validated using a biomechanical model.

### 2.7 Relationships with disease

Differences in functional metrics between the reference group and each disease category were tested using the Wilcoxon rank sum test. To examine the discriminative ability of the PC scores compared with standard functional metrics, linear discriminant analysis models were constructed for each prevalent disease category. In each model, positive cases were those with the disease and negative cases were those in the disease-free reference cohort. For each disease category, two linear discriminant analysis model were compared, one in which the discriminatory variables were PC scores and the other in which the discriminatory variables comprised LV and RV end-diastolic and end-systolic volumes, ejection fractions, global circumferential and longitudinal strains, and MAPSE and TAPSE. The area under the curve (AUC) was compared between the two models using the DeLong test. For this comparison, the AUCs were constructed using the test cases from a five-fold cross validation analysis using the R caret package (version 6.0–93) [35]. An additional comparison was performed with covariates age, sex, body mass index, height, and afterload added to the discriminatory variables of both linear discriminant analysis models.

Although the PC scores are uncorrelated by definition, multicollinearity may exist between the standard measures (volume and strains). Multicollinearity can have a large effect on the regression parameters (betas), but may not change the overall AUC greatly. This was tested by successively excluding variables with variance inflation factor (VIF) greater than 5, until all variables had VIF<5 [50]. If both LV and RV measures of volume had VIF>5, the RV measure was removed first.

To test the benefit of adding standard variables to PC scores, we also compared PCs plus standard variables plus covariates against standard variables plus covariates, removing variables with VIF>5.

### 2.8 Manual volume and tagging analysis

Volumes and strain results from the automated analyses were compared with manual analyses previously performed in the first 5000 CMR imaging participants [36], [37]. The manual volume analysis was performed by drawing short axis ventricular contours and valve landmarks, using cvi42. Calculation of volumes and mass was performed using slice summation in cvi42 [36]. Manual strain analysis of the three short axis slices acquired with spatial modulation of magnetization (SPAMM) tissue tagging was performed using CIMTag2D v8.1.5 software, (Auckland MRI Research Group, New Zealand), which has been validated previously in phantoms and patients [38]. As previously described [37], a tag grid was aligned automatically to the myocardial tagging planes at end-diastole. End-systole was determined visually, and tag tracking was performed using nonrigid image registration, with manual adjustments at key phases during the cardiac cycle including the end-systolic and late diastolic frames. Circumferential myocardial strain was calculated by the software from the motion of the tag lines, averaged over the whole slice.

## 3 Results

### 3.1 Study population, quality control and automated analysis

Of 45,683 CMR examinations available for analysis at the time of study, 3D shape models could be customized at ED and ES in 43,159 cases (Fig. 1A, B, C). PCA was performed on 41,659 cases (Fig. 1C). Model quality control (QC) resulted in 1500 cases rejected before the PCA. After PCA, 1261 cases were excluded for high Mahalanobis distance, 463 cases for high PC projection error, and 1832 cases for volume differences from cvi42 (the total 2801 in Fig. 1E is due to many cases being rejected for multiple criteria). After QC, there was a total of 38,858 cases, 5149 with prevalent cardiovascular or cardiometabolic disease and 33,709 without.

Table 1 shows participant demographics, comparing the reference sub-cohort with the disease sub-cohorts. In general, those with disease were older, more likely male, with higher blood pressure and body mass index. Table 1 also shows ventricular volumes and LV mass computed from the shape models by numerical integration. Those with disease had significantly higher left and right ED and ES volumes, higher LV mass, and lower right and left EF. The reference group volumes and mass were within the reference range for normal cardiac structural and functional measures detailed previously [36].

**Table 1:** Participant characteristics for sub-cohorts with and without disease.

Characteristic	Disease (n = 5149)	Reference (n = 33,709)[1]
Age (years)	67 (7)	63 (8)*
Sex (male)	3,453 (67%)	14,974 (44%)*
Weight (kg)	83 (15)	75 (14)*
Height (cm)	171 (9)	169 (9)*
BMI (kg/m^2^)	28.2 (4.6)	26.3 (4.0)*
SBP (adjusted)	147 (21)	138 (20)*
DBP (adjusted)	86 (12)	82 (11)*
LV EDV (mL)	155 (36)	146 (32)*
LV ESV (mL)	74 (22)	67 (18)*
RV EDV (mL)	153 (35)	147 (35)*
RV ESV (mL)	70 (20)	65 (20)*
LV Mass (g)	131 (27)	123 (26)*
LVEF (%)	52.4 (6.4)	54.2 (4.8)*
RVEF (%)	54.7 (6.7)	56.1 (5.8)*
Heart failure	341 (6.6%)	.
Atrial fibrillation	1,088 (21%)	.
Myocardial infarction, ischemic heart disease	2,389 (46%)	.
Hypertrophic cardiomyopathy	26 (0.5%)	.
Dilated cardiomyopathy	32 (0.6%)	.
Diabetes mellitus	1,656 (32%)	.
Ventricular arrhythmia composite	151 (2.9%)	.
Conduction disease	635 (12%)	.

Values are mean (standard deviation) for continuous variables and count (%) for categorical variables. Conduction disease: bundle branch block, fascicular block or atrioventricular block. Ventricular arrhythmia composite: ventricular arrhythmia, cardiac arrest, sudden cardiac death, defibrillator implantation). All variables were significantly different between Reference and Disease groups. *LV* left ventricle, *EDV* end-diastolic volume, *BMI* body mass index, *SBP* systolic blood pressure, *DBP* diastolic blood pressure, *ESV* end-systolic volume, *EF* ejection fraction

* p<0.001 Disease vs Reference, Wilcoxon rank sum test

### 3.2 Strain, MAPSE and TAPSE

Table 2 shows global geometric longitudinal and circumferential strain computed from model arc lengths, and MAPSE and TAPSE computed from the displacement of model valve points, in reference and disease groups. Compared to the reference group, most strain measures were significantly (p < 0.05) reduced in all disease groups, except for hypertrophic cardiomyopathy in both LV GCS (mid) and RV GCS (mid), ventricular arrhythmia composite in RV GCS (mid) and RV GLS, and diabetes in RV GLS. MAPSE and TAPSE were also reduced in all disease groups compared to the reference (p < 0.05).

**Table 2:** Geometric strain (% shortening) and MAPSE/TAPSE in different disease categories.

Group	LV GCS (mid)	LV GLS	RV GCS (mid)	RV GLS	MAPSE	TAPSE
Reference	27.0 (3.6)	20.6 (2.6)	23.8 (5.6)	32.1 (5.3)	12.2 (2.2)	17.6 (3.3)
Atrial fibrillation	25.3 (5.2)†	18.6 (3.9)†	22.5 (6.1)†	29.4 (6.8)†	10.9 (2.8)†	15.4 (4.5)†
Dilated cardiomyopathy	19.6 (4.9)†	15.8 (3.6)†	17.7 (6.3)†	25.5 (6.7)†	9.7 (2.4)†	14.1 (3.8)†
Diabetes mellitus	26.3 (4.4)†	19.9 (3.0)†	22.7 (6.0)†	31.9 (5.9)	11.1 (2.3)†	16.0 (3.8)†
Hypertrophic cardiomyopathy	26.1 (6.0)	17.8 (3.5)†	23.6 (6.8)	28.1 (6.2)*	10.6 (3.3)*	14.9 (4.4)†
Heart failure	23.4 (5.3)†	17.8 (3.7)†	22.2 (6.2)†	30.0 (6.6)†	10.4 (2.6)†	15.2 (4.1)†
Myocardial infarction, ischemic heart disease	25.9 (4.6)†	19.5 (3.1)†	23.1 (5.9)†	31.3 (5.9)†	11.4 (2.3)†	16.1 (4.0)†
Conduction disease	24.8 (4.6)†	19.0 (3.1)†	22.7 (6.1)†	30.3 (6.2)†	11.4 (2.4)†	16.0 (3.8)†
Ventricular arrhythmia composite	25.0 (5.2)†	18.7 (3.7)†	22.9 (5.0)	30.9 (6.2)	11.2 (2.4)†	16.4 (4.0)†

Mean (standard deviation). *GCS* global circumferential strain, *GLS* global longitudinal strain, *MAPSE* mitral annular plane systolic excursion, *TAPSE* tricuspid valve plane annular excursion, *LV* left ventricle, *RV* right ventricle. Wilcoxon rank sum test disease vs Reference

† P<0.001

* P<0.05

**Fig. 2:**
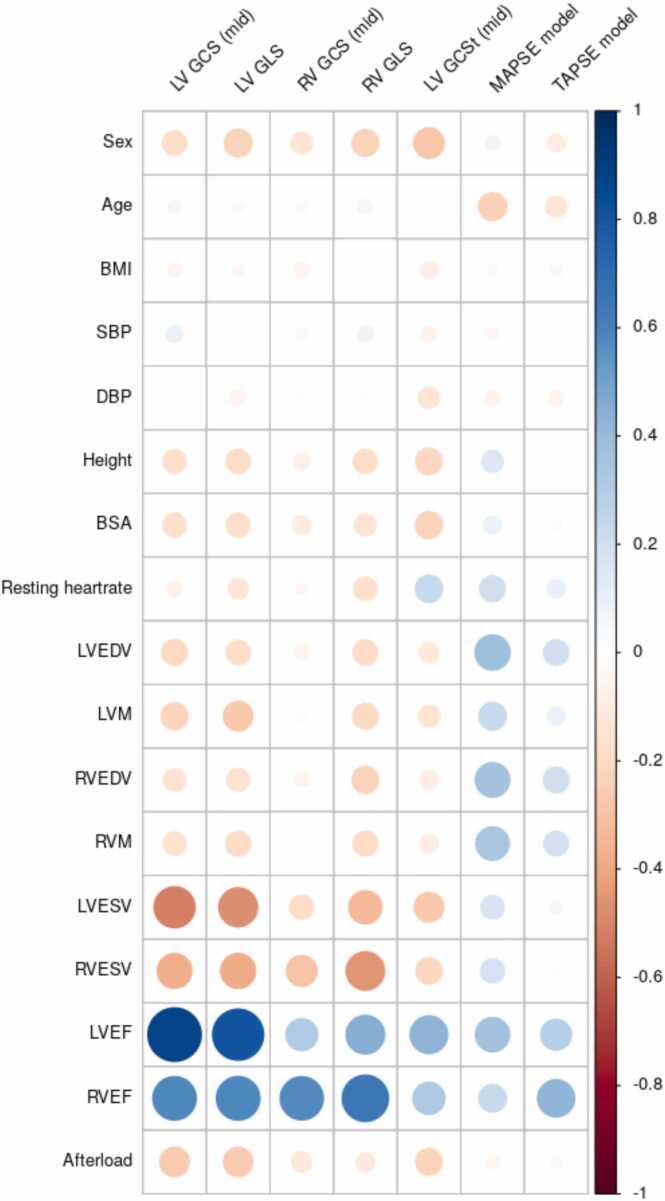
Univariate correlations between strain and covariates in the reference cohort. *LV* left ventricular, *RV* right ventricular, *GCS* global circumferential strain, *GLS* global longitudinal strain, *MAPSE* mitral annular plane systolic excursion, *TAPSE* tricuspid annular plane systolic excursion, *BMI* body mass index, *SBP* systolic blood pressure adjusted for presence of medication, *DBP* diastolic blood pressure adjusted for presence of medication, body surface area, body surface area, *EDV* end-diastolic volume, *ESV* end-systolic volume, *M* mass, *EF* ejection fraction. Model strains and MAPSE/TAPSE from n=33709 cases; tagging strain from n=3280 cases

Fig. 2 shows univariate correlations between standard functional metrics and covariates within the reference group. Strain was decreased in males, and also with increased height, body surface area, ventricular volume, LV mass, and LV afterload. Strain was not as strongly correlated with age, body mass index, or blood pressure. In contrast, MAPSE and TAPSE increased with height, body surface area, larger ventricular volume and LV mass, and was not as strongly correlated with sex, body mass index, and afterload. Multivariate regression results are presented in Supplementary Table S1.

### 3.3 Principal components

**Fig. 3:**
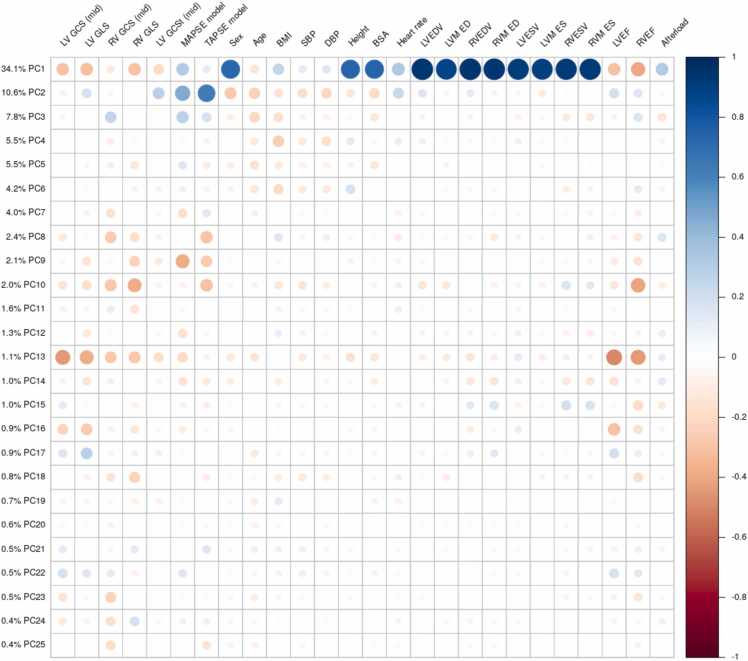
Univariate correlations between PCs and demographics, volumes, and strain. The amount of shape variance explained by each PC is shown on the left. *LV* left ventricular, *RV* right ventricular, *GCS* global circumferential strain, *GLS* global longitudinal strain, *GCSt* global circumferential strain (tagging), *MAPSE* mitral annular plane systolic excursion, *TAPSE* tricuspid annular plane systolic excursion, *BMI* body mass index, *SBP*, systolic blood pressure adjusted for presence of medication, *DBP* diastolic blood pressure adjusted for presence of medication, *BSA* body surface area, *EDV* end-diastolic volume, *ESV* end-systolic volume, *M* mass, *EF* ejection fraction. All correlations N=33,709 cases except tagging N=3,280. Supplemental Fig. S1 ROC curves for linear discriminant analyses (Table 3). Blue: PC model; Green: standard model. Supplemental Fig. S2 Bland-Altman Plots: Manual vs Shape Model

We selected the first 25 PCs of shape variation, which represent the largest modes of variation of biventricular shape within the cohort, and together accounted for over 90% of the total shape variance. Fig. 3 shows the amount of shape variance explained by each PC. Animations of these can be found in Supplemental Movies. Since these modes result from unsupervised dimension reduction, anatomical interpretation is typically not possible; however, clear interpretations can be made for some modes. In particular, PC1 (34% of shape variance) was associated with overall size at both ED and ES with positive scores associated with larger hearts. PC2 (11%) was associated with systolic motion, with positive scores associated with larger displacement, in particular basal descent of the valves during systole (TAPSE and MAPSE). PC3 (8%) was associated with sphericity of both ventricles at ED and ES, with positive scores related to less spherical shapes. PC9 (2.1%) was associated with systolic basal excursion, PC10 (2%) was associated with sphericity of the right ventricle, and PC13 (1.1%) was associated with ejection fraction.

Fig. 3 shows univariate correlations between PCs and demographics, volumes, and strain. PC1 (overall size) was positively correlated with ventricular volumes and mass, male sex, height and body surface area, afterload, and MAPSE and TAPSE. PC1 was negatively correlated with strain and EF. PC2 (longitudinal motion of the valves) was positively correlated with MAPSE and TAPSE, and to a lesser extent strain and EF. PC3 (sphericity) was correlated with RV GCS and MAPSE/TAPSE. PC 9 was correlated with MAPSE/TAPSE and to a lesser extent strain. PC10 was correlated with RV strain and EF, as well as TAPSE. PC13 was correlated with LV and RV strain and EF, and to a lesser extent MAPSE and TAPSE.

## 4 Relationships with disease

Strengths of relationships with cardiovascular or cardiometabolic diseases are shown in Table 3. Receiver operating characteristic curves are shown in Supplementary Fig. S1. Comparing linear discriminant analysis models using the first 25 shape PC scores as discriminatory variables against a “standard” model with volumes, mass, ejection fraction, strain, MAPSE and TAPSE as discriminatory variables, the shape scores had significantly stronger relationships with prevalent atrial fibrillation, heart failure, myocardial infarction or ischemic heart disease, diabetes and conduction disease. Relationships with hypertrophic and dilated cardiomyopathies and ventricular arrhythmia were similar between metrics, however the disease cohorts for these groups were relatively small (n=26, 32 and 152, respectively). When covariates age, sex, body mass index, height, and afterload were added to the discriminatory variables of both models, AUC improved and differences between models were reduced, as expected since the covariates are strongly related to disease and mask the differences between shape scores and strain/volume measures (Table 4). However, relationships with shape were still significantly stronger than those with volumes and strain (DeLong p < 0.05) in atrial fibrillation, heart failure, diabetes and conduction disease (Table 4).

**Table 3:** Comparison of linear discriminant analysis AUC of disease prevalence: PC scores vs standard metrics.

Disease	AUC		DeLong test P-value
	PCs (1-25)	Standard	
Atrial fibrillation (n=1,088)	0.76	0.71	8.9E−13
Heart failure (n=341)	0.82	0.78	2.4E−05
Hypertrophic cardiomyopathy (n=26)	0.79	0.83	0.14
Dilated cardiomyopathy (n=32)	0.92	0.91	0.70
Ventricular arrhythmia composite (n=151)	0.73	0.68	0.04
Myocardial infarction or ischemic heart disease (n=2389)	0.73	0.69	1.0E−20
Diabetes mellitus (n=1,656)	0.77	0.68	2.1E−56
Conduction disease (n=635)	0.78	0.74	6.4E−08

Disease: cardiovascular or cardiometabolic disease; *AUC* area under the curve, *PCs* principal components. *LV* and *RV* end-diastolic and end-systolic volumes, ejection fractions, global circumferential and longitudinal strains, *LV* mass, and *MAPSE* and *TAPSE* are all included in the Standard model

**Table 4:** Comparison of linear discriminant analysis AUC of CMD prevalence, including covariates in both models.

CMD	AUC		DeLong test P-value
	PCs	Standard	
Atrial fibrillation (n=1,088)	0.77	0.76	1.8E−03
Heart failure (n=341)	0.85	0.82	4.9E−04
Hypertrophic cardiomyopathy (n=26)	0.82	0.83	0.55
Dilated cardiomyopathy (n=32)	0.94	0.89	0.10
Ventricular arrhythmia (composite) (n=151)	0.74	0.72	0.19
Myocardial infarction or ischemic heart disease (n=2,389)	0.77	0.77	0.18
Diabetes mellitus (n=1,656)	0.82	0.81	2.2E−04
Conduction disease (n=635)	0.81	0.79	4.9E−04

*CMD* cardiometabolic disease, *AUC* area under the curve, *PCs* principal components. Standard: LV and RV end-diastolic and end-systolic volumes, ejection fractions, global circumferential and longitudinal strains, LV mass, and MAPSE and TAPSE. Covariates were age at imaging, sex, height, weight, BMI and afterload

To assess the effects of multicollinearity, variance inflation factors (VIF) were computed for the discriminator variables in all models. Those with VIF>5 were successively removed. For the PC models, no discriminator variables needed to be removed. For the standard model without covariates, RV end-diastolic and end-systolic volume, LV end-systolic volume, and LV ejection fraction were removed. Results are shown in Table S2, and were very similar to Table 3. For the standard model with covariates, RV end-diastolic and end-systolic volume, LV end-diastolic volume and end-systolic volume, and LV ejection fraction were removed (none of the age, sex, height, weight, body mass index (BMI) and afterload covariates had VIF > 5). Results are shown in Table S3, and were very similar to Table 4.

We also evaluated the effect of adding standard variables to the PC scores and covariates, compared with the standard variables and covariates. Since standard volume and strain measures are correlated with PCs, those with VIF>5 were removed. For the PC model, all PCs, LV and RV global circumferential and longitudinal strains, and all covariates age, sex, height, weight, BMI and afterload, remained after removal of variables with VIF >5. Results are shown in Table S4, and were very similar to Table 4, and Table S3. This indicates that adding standard functional measures to the PCs has little effect on discriminatory power of the PC scores.

### 4.1 Manual volumes and tagging strain

Within the 5000 participants who had manual volume analysis, 4257 had both manual and automatic shape model estimates of mass and volume (669 disease and 3588 reference). Comparisons between manual and shape model estimates for disease and reference cases within this sub-cohort are shown in Table 5. In general, the LV volumes and mass computed from the shape models using numerical integration were larger than the manual estimates. This is likely due to differences in computation methods, with the model volume and mass including structures up to the valves and at the apex tip whereas manual estimates were performed with short axis slices only using slice summation. LV mass was also larger in the model estimates, partly due to allocation of mass to the LV at the junction of the RV free wall and the septum in the model, which is excluded in standard LV contours [39]. The differences in volume and mass were consistent between disease and reference cases for both methods (Table 5). Bland-Altman plots of differences between methods are shown in Supplemental Material.

**Table 5:** Model and manual analyses of volume and strain.

	Shape Model		Manual	
Characteristic	Disease (n=669)	Reference (n=3,588)	Disease (n=669)	Reference (n=3,588)
LVEDV (mL)	157 (37)*	148 (32)	151 (37)*	142 (32)
LVESV (mL)	75 (23)*	68 (18)	63 (23)*	58 (17)
LVM (g)	132 (28)*	123 (26)	99 (26)*	87 (23)
RVEDV (mL)	154 (36)*	148 (35)	158 (37)*	151 (37)
RVESV (mL)	69 (21)*	65 (20)	70 (23)*	67 (22)
LV GCS (mid, %)^1^	26.2 (4.1)*	27.2 (3.3)	21.5 (3.3)*	22.5 (2.9)

Mean (standard deviation); Disease vs Reference, Wilcoxon rank sum test; ^1^Strain for n=565 for disease and 3280 for reference cases with both manual tagging (midventricular) and model strain. *LV* left ventricle, *RV* right ventricle, *EDV* end-diastolic volume, *ESV* end-systolic volume, *LVM* left ventricular mass, *GCS* global circumferential strain

* p<0.001

Within the first 5000 participants who had manual tagging strain analysis, 3845 had both manual and automatic shape model strain estimations. Geometric circumferential mid-ventricle strains and tagging strains are shown in Table 5 for each sub-cohort. Bland-Altman plots are shown in the Supplemental Materials. LV global circumferential midventricular strain was higher than tagging circumferential strain, which was expected since endocardial circumferential strain is typically higher than mid-wall and epicardial strain (the tagging estimate being an average over the whole wall) [40]. Differences between disease and reference cohorts were consistent between shape model and tagging strain estimates. Correlations between tagging GCS and covariates (sex, height, afterload, LV volume etc) followed similar patterns to geometric model GCS (Fig. 2). Correlations between tagging GCS and PCs (Fig. 3) also followed similar patterns to geometric model GCS.

## 5 Sensitivity of combined metrics

Disease-specific combined PC scores can be used to quantify functional impairment and track changes with disease development or treatment effects. For example, the standardized combined z-score obtained from the linear discriminant analysis models is the linear combination of PC scores which best discriminate the presence of disease. Table 6 shows the disease-specific combined z-scores for each disease category. These disease-specific z-scores are intuitive, in that reference (healthy) cases have a mean around zero and a standard deviation around one, and disease cases have a higher score in units of standard deviations. The differences in z-scores between reference and disease cases are more highly significant for shape scores than standard metrics in all disease categories. Studies designed to detect a change in heart function with disease progression or treatment would therefore have more power to detect an effect. For example, in diabetes the z-scores were 0.00 ± 0.97 (reference) vs 0.99 ± 1.00 (diabetes) for PCs and −0.03 ± 0.98 vs 0.68 ± 1.15 for standard metrics. A power calculation of the number of participants needed for a study of heart function in diabetes would require 32 participants (16 in each group) to detect a difference between patients and controls using PC scores, and 68 participants (34 in each group) for standard metrics, assuming an alpha of 0.05, power of 0.8.

**Table 6:** Linear discriminant scores for each disease category (z-scores).

	PCs (1−25)			Standard		
Disease	Reference	Disease	P-value	Reference	Disease	P-value
Atrial fibrillation (n=1088)	−0.04±0.95	1.32±1.59	9.8E−132	−0.03±0.97	0.99±1.43	1.2E−98
Heart failure (n=341)	−0.02±0.98	1.79±1.58	7.2E−64	−0.02±0.95	1.94±2.69	1.8E−33
Hypertrophic cardiomyopathy (n=26)	−0.00±1.00	2.25±1.63	2.1E−07	−0.00±1.00	2.08±1.46	1.2E−07
Dilated cardiomyopathy (n=32)	−0.00±0.99	3.05±1.56	2.6E−12	−0.00±0.99	3.97±3.59	6.1E−07
Ventricular arrhythmia composite (n=151)	−0.01±0.99	1.27±1.57	2.4E−18	−0.01±0.98	1.56±3.06	4.2E−09
Myocardial infarction or ischemic heart disease (n=2,389)	−0.07±0.94	0.93±1.26	2.9E−251	−0.05±0.93	0.79±1.47	3.9E−148
Diabetes mellitus (n=1,656)	−0.05±0.97	0.99±1.00	3.3E−264	−0.03±0.98	0.68±1.15	4.3E−117
Conduction disease (n=635)	−0.02±0.98	1.30±1.36	1.2E−93	−0.02±0.96	1.22±1.84	1.7E−53

Data are means +/- standard deviation. Disease: cardiovascular or cardiometabolic disease; *PCs* principal components, Standard: LV and RV end-diastolic and end-systolic volumes, ejection fractions, global circumferential and longitudinal strains, LV mass, and MAPSE and TAPSE

## 6 Discussion

Evaluation of cardiac disease typically includes functional assessment including ejection fraction, strain, and valve plane motion, commonly assessed with echocardiography or CMR [1], [41]. However, these metrics do not capture all the shape change information present in modern medical imaging examinations. Here, we showed that shape scores derived from a statistical shape atlas, including both ED and ES shape information, are more strongly discriminative of disease than standard volumetric, strain and valve motion metrics (Table 3). The PC scores can be automatically calculated at the time of imaging (Fig. 1), by segmenting the images, customizing a shape model, and computing the PC scores. Combined PC scores (such as linear discriminant analysis z-scores in Table 6) can be used to quantify patient status relative to a reference cohort, such as that provided in the UK Biobank. This provides a potentially more powerful set of functional metrics than is currently available, enables more sensitive quantification of disease effects, and can reduce the number of subjects required for studies of disease status and treatment effects.

In particular, PC scores performed well for discrimination of diabetes mellitus, myocardial infarction, conduction disease, atrial fibrillation and heart failure, suggesting PC scores contain more information on shape and shape changes during systole in these disease categories. No significant differences were found for hypertrophic and dilated cardiomyopathies, with relatively high AUC for both sets of discriminatory variables, indicating that traditional measures are very discriminative for these diseases. However, the number of hypertrophic and dilated cardiomyopathies cases was low in this cohort and the method should be tested in larger disease cohorts. Ventricular arrhythmia discrimination was also similar between shape scores and traditional metrics, with moderate AUC, which may indicate that systolic shape changes are not as informative in these pathologies.

Our pipeline for atlas shape quantification was fully automated, with some similar characteristics to previously published automated analysis pipelines for quantification of cardiac shape and function [23], [24], [25], [26], [42], [43], [44]. However, to our knowledge the current study is the first to compare PC shape scores with EF, strain and MAPSE/TAPSE functional indices for the discrimination of disease.

Geometric strain computed from arc lengths had good agreement with tagging strain, as found in previous studies [31], [32]. As expected, endocardial strain was higher than tagging mid-wall strain. Strain was significantly decreased with most examined diseases (Table 2), consistent with previous studies [1]. In particular, a previous study of 3984 UK biobank participants showed reduced tagging strain in 143 diabetes mellitus participants [45]. The current study corroborates this finding, with the diabetes mellitus group (n=1656) having reduced geometric strain for both LV and RV GCS and GCS, as well as MAPSE and TAPSE.

There was a significant dependence of strain on afterload, in agreement with previous studies [10], [46]. Also, strain was reduced with male sex and increased body size (body surface area and height), in agreement with previous studies [24], [41], [43], [47]. Some previous studies have shown that MAPSE may have different prognostic information to strain [7], [8] and has different relationships with body size than strain [11]. Here, MAPSE had different relationships with age, body size and afterload to strain, with reduced dependence on afterload and body size, but stronger dependence on age. Moreover, some PC scores were highly correlated with MAPSE and TAPSE (PC2 and PC9 in Fig. 3) whereas others were more correlated with strain (PC10 for RV strain and PC13 for LV strain), consistent with strain and MAPSE information being captured in the PC scores. Correlations between PC scores and sex, height and afterload were also found, particularly with PC1 which was associated with overall heart size. Other PCs had greatly reduced correlations with body habitus and afterload, including PC2 (MAPSE) and PC10 and PC13 (RV and LV strain).

## 7 Limitations

Limitations of this study include use of UK Biobank data, which comprised mainly low-risk participants with selection bias arising from volunteering for the study. Subsequently there is a lack of cases with disease in cardiomyopathy and arrhythmia categories. Although an automated quality control procedure was used to remove outliers, it is likely that additional QC of the automated pipeline including analysis of all frames in the cine sequence such as in Ruijsink *et al.,* 2020 [42] would benefit results. Mistakes in automatic segmentation will lead to errors in the PC scores, although the first 25 scores used in this study are relatively robust to small errors in segmentations. Disease was classified by self-reported incidence or hospital electronic health records and may not fully capture the diseases present in individuals in early asymptomatic stages of disease. Co-morbidities between diseases were not considered since the nature of the relationships between shape score and disease require a detailed analysis of how shape varies with height, sex, age, and co-morbidities, which will require a larger disease cohort (UK Biobank will shortly have 100,000 imaging participants, enabling some disentanglement of these effects). Table 4 shows only modest AUC improvements when covariates age, sex, body mass index, height, and afterload are included in the discrimination of atrial fibrillation, heart failure, diabetes and conduction disease. This is due to these covariates being highly discriminative, masking differences between PC and standard heart function measures. However, the main utility lies in improved sensitivity of z-scores derived from PCs (Table 6), e.g. enabling 50% fewer diabetic patients to detect differences from a reference group, thereby facilitating studies of mechanistic effects of disease and treatment. LV volume and mass were significantly higher in the models compared to standard slice summation (Table 5). This is likely due to systematic differences in calculation methods, with numerical integration of model mass including muscle up to the RV free wall, and up to the valve locations. Finally, a recent study of 45,700 UK Biobank participants showed feature tracking strain was independently predictive of incident heart failure, myocardial infarction and stroke [48]. Having established improved sensitivity with prevalent disease, future work should study the utility of atlas scores in predicting future adverse events, and assess their utility combined with other data from applicable examinations, e.g. LGE and mapping information, stress perfusion data, calcium scores, aortic distensibility, etc. Such predictive models will need to examine the multicollinearity between predictors seen in Fig. 2 and Fig. 3. Two previous methods used for incident disease prediction include partial least squares [22] and linear discriminant analysis with careful feature selection [49]. The examination of changes in disease-specific scores (such as in Table 6) should also be extended to longitudinal studies.

## 8 Conclusions

Atlas-based shape and motion scores capture more of the available shape change information present in modern imaging examinations than standard measures of cardiac function. An automated analysis pipeline enables routine evaluation of disease-specific z-scores at the time of imaging. Atlas scores provide more sensitive metrics for the evaluation of disease effects.

## Funding

R.B. and A.Y. acknowledge core funding from the Wellcome/EPSRC Centre for Medical Engineering [WT203148/Z/16/Z] and The London Medical Imaging & AI Centre for Value Based Healthcare. A.Y. acknowledges funding from the National Institutes of Health (USA) R01HL121754. S.E.P. and P.B.M. acknowledge support from the National Institute for Health and Care Research (NIHR) Biomedical Research Centre at Barts (NIHR202330). J.R. acknowledges funding from fellowship RYC2021–031413-I from 10.13039/501100004837Spanish Ministry of Science and Innovation.

## Author contributions

R.B., S.E.P., J.R., P.M. and A.Y. designed the study. A.Y., L.D.T., C.M., A.S. developed the methods. A.S. supervised the Circle analyses. R.B. and A.Y. performed the model analyses and drafted the manuscript. All authors participated in revision and read and approved the final manuscript.

## Ethics approval and consent

UK Biobank was approved by National Research Ethics Service North West (11/NW/0382). All participants gave written informed consent.

## Consent for publication

No identifiable individual information was included in this study.

## Declaration of competing interests

The authors declare the following financial interests/personal relationships which may be considered as potential competing interests Alireza Sojoudi reports a relationship with Circle Cardiovascular Imaging Inc that includes: employment. Steffen Petersen reports a relationship with Circle Cardiovascular Imaging Inc that includes: consulting or advisory. If there are other authors, they declare that they have no known competing financial interests or personal relationships that could have appeared to influence the work reported in this paper.

## Data Availability

This research has been conducted using the UK Biobank resource under application 2964. The raw data, the derived data, the analysis and results have been clearly annotated and returned to UK Biobank, which will then incorporate the returned data into the central repository. UK Biobank will make the data available to all bona fide researchers for all types of health-related research that is in the public interest, without preferential or exclusive access for any person. All researchers will be subject to the same application process and approval criteria as specified by UK Biobank. For the detailed access procedure see http://www.ukbiobank.ac.uk/register-apply/.

## Footnotes

Appendix A Supplementary data associated with this article can be found in the online version at doi:10.1016/j.jocmr.2025.101919.
