# Supplementary material for: Relationships between heart shape, function, and disease in 38,858 UK biobank participants
Source: J Cardiovasc Magn Reson. 2025 Jun 2;27(2):101919. doi: 10.1016/j.jocmr.2025.101919 (PMC12780292; doi:10.1016/j.jocmr.2025.101919)
Supplement: Supplementary file 1 — Supplementary material [file mmc1.pdf]

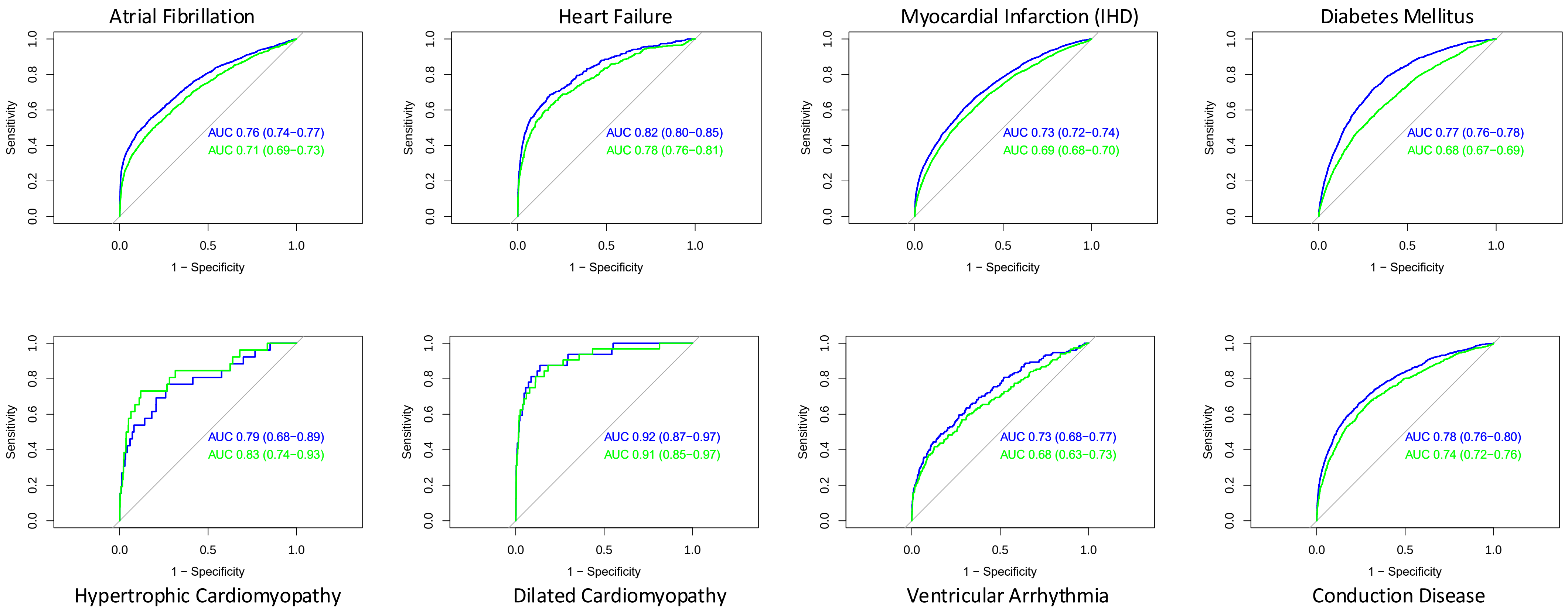

Supplemental Fig S1. ROC curves for linear discriminant analyses.

Blue: PC model; Green: standard model.
