# Supplementary material for: Relationships between heart shape, function, and disease in 38,858 UK biobank participants
Source: J Cardiovasc Magn Reson. 2025 Jun 2;27(2):101919. doi: 10.1016/j.jocmr.2025.101919 (PMC12780292; doi:10.1016/j.jocmr.2025.101919)
Supplement: Supplementary file 2 — Supplementary material [file mmc2.docx]

**Supplementary Material**

**Quality Control (QC)**

*Data QC:* Image contours and landmarks were required to contain sufficient 3D information to create shape models at the ED and ES frames (Figure 1C). Three or more short axis slices were required per case. A complete set of 10 valve points (6 mitral comprising 2 from the two-chamber, 2 from the three-chamber, 2 from the four-chamber; 2 aortic from the three-chamber; 2 tricuspid from the four-chamber) was required at ED and ES.

*Model QC:* Model outliers in which the volumes of any chamber exceeded 5 inter-quartile range (IQR) at ED and ES were excluded prior to the PCA. Models in which the computed distance between the surface of the fitted model and the guide points exceed 5 IQR were also excluded (Figure 1D).

*Post PCA QC:* A set of criteria were imposed on the models after PCA:

1. Mahalanobis distance- we excluded outliers exceeding 3 IQR in Mahalanobis distance applied over the first 10 PCA modes.
2. PC projection- we also computed the error in mm between each individual shape and its projection onto the first 10 PCA modes, to provide an estimate of the difference between the shape and its low dimension representation. This provided an estimate of residual distance uncaptured by the Mahalanobis distance, and outliers exceeding 3 IQR were excluded.
3. Volume error- models were excluded in which the difference between left or right ventricular volume at ED or ES computed from the model by numerical integration and the volume calculated automatically from the cvi42 software from the contours exceeded 3 IQR.

**Strain and MAPSE/TAPSE Multivariate Regression**

Multivariate regressions of automatically derived strain against covariates in in the reference sub-cohort (Table S1) showed similar relationships as univariate relationships. Strain remained significantly decreased with increased afterload and male sex, when controlling also for age, height and BMI. Strain remained increased with increased age for LV GCS and GLS, tagging, and RV GLS, but this was not significant with RV GCS. LV GCS and GLS, and RV GCS, remained significantly reduced with increasing height, but this was not significant in RV GLS and tagging. Relationships with BMI were typically reduced from univariate values, but remained significant in LV GLS, RV GCS, RV GLS and tagging.

MAPSE and TAPSE remained decreased with increasing age and BMI in multivariate regressions (Table S1) with strongly significant increases with height. Relationships with afterload remained weak. TAPSE remained decreased with male sex, but this was not significant for MAPSE.

## Table S1. Multivariate regressions of strain measures against risk factors (reference sub-cohort). P values of 0 are within the bounds of machine precision from R. Predictors were standardized; response variable was not. GCS, global circumferential strain; GLS, global longitudinal strain; GCS tagging, global circumferential strain from manual tagging analysis (1N= 3,280); MAPSE, mitral annular plane systolic excursion; TAPSE, tricuspid annular plane systolic excursion; BMI, body mass index.

|  | Beta | | | | | P-value | | | | |
| --- | --- | --- | --- | --- | --- | --- | --- | --- | --- | --- |
|  | **Age** | **Sex** | **BMI** | **Height** | **Afterload** | **Age** | **Sex** | **BMI** | **Height** | **Afterload** |
| LV GCS (mid) | 0.41±0.02 | -0.29±0.03 | 0.02±0.02 | -0.23±0.03 | -0.94±0.02 | 9.7E-99 | 3.6E-25 | 0.25 | 1.5E-16 | 0 |
| LV GLS | 0.06±0.01 | -0.42±0.02 | 0.07±0.01 | -0.07±0.02 | -0.60±0.01 | 3.5E-05 | 4.9E-100 | 6.3E-07 | 3.8E-04 | 0 |
| RV GCS (mid) | -0.03±0.3 | -0.78±0.04 | -0.19±0.03 | 0.18±0.04 | -0.50±0.03 | 0.39 | 1.0E-67 | 2.6E-09 | 6.9E-05 | 3.0E-54 |
| RV GLS | 0.51±0.03 | -1.1±0.04 | 0.20±0.03 | -0.09±0.04 | -0.45±0.03 | 2.4E-69 | 6.3E-143 | 4.6E-12 | 0.034 | 7.8E-51 |
| LV GCS tagging (mid)^1^ | 0.18±0.05 | -0.65±0.07 | -0.11±0.05 | -0.08±0.07 | -0.49±0.05 | 3.7E-04 | 8.2E-20 | 0.036 | 0.28 | 1.9E-20 |
| MAPSE | -0.49±0.01 | -0.02±0.02 | -0.06±0.01 | 0.33±0.02 | -0.05±0.01 | 0 | 0.14 | 2.1E-06 | 2.4E-86 | 8.6E-06 |
| TAPSE | -0.42±0.02 | -0.62±0.03 | -0.09±0.02 | 0.42±0.03 | 0.07±0.02 | 3.8E-116 | 1.9E-120 | 3.6E-07 | 2.5E-58 | 9.5E-05 |

Table S2. Comparison of linear discriminant analysis AUC of disease prevalence: PC scores vs standard metrics, after removal of variables with variance inflation factor > 5. Disease: cardiovascular or cardiometabolic disease; AUC, area under the curve; PCs: principal components; Standard: LV end-diastolic volume, LV mass, RV ejection fraction, LV and RV global circumferential and longitudinal strains, and MAPSE and TAPSE.

| Disease | AUC | | DeLong test P-value |
| --- | --- | --- | --- |
|  | **PCs (1-25)** | **Standard** |  |
| Atrial fibrillation (n=1088) | 0.76 | 0.71 | 2.6E-11 |
| Heart failure (n=341) | 0.82 | 0.79 | 8.3E-04 |
| Hypertrophic cardiomyopathy (n=26) | 0.79 | 0.83 | 0.28 |
| Dilated cardiomyopathy (n=32) | 0.92 | 0.92 | 0.93 |
| Ventricular arrhythmia composite (n=151) | 0.73 | 0.71 | 0.31 |
| Myocardial infarction or ischaemic heart disease (n=2389) | 0.73 | 0.69 | 1.5E-18 |
| Diabetes mellitus (n=1656) | 0.77 | 0.67 | 1.2E-60 |
| Conduction disease (n=635) | 0.78 | 0.74 | 2.2E-08 |

Table S3. Comparison of linear discriminant analysis AUC of CMD prevalence, including covariates in both models, after removal of variables with variance inflation factor > 5. CMD, cardiometabolic disease; AUC, area under the curve; PCs: principal components. Standard model: LV mass, RV ejection fraction, LV and RV global circumferential and longitudinal strains, and MAPSE and TAPSE. Covariates were: age at imaging, sex, height, weight, BMI and afterload (none removed).

| CMD | AUC | | DeLong test P-value |
| --- | --- | --- | --- |
|  | **PCs** | **Standard** |  |
| Atrial fibrillation (n=1088) | 0.77 | 0.75 | 9.8E-04 |
| Heart failure (n=341) | 0.85 | 0.83 | 4.8E-03 |
| Hypertrophic cardiomyopathy (n=26) | 0.82 | 0.83 | 0.77 |
| Dilated cardiomyopathy (n=32) | 0.94 | 0.92 | 0.45 |
| Ventricular arrhythmia (composite) (n=151) | 0.74 | 0.73 | 0.60 |
| Myocardial infarction or ischaemic heart disease (n=2389) | 0.77 | 0.77 | 0.47 |
| Diabetes mellitus (n=1656) | 0.82 | 0.80 | 9.6E-12 |
| Conduction disease (n=635) | 0.81 | 0.79 | 5.7E-04 |

Table S4. Comparison of PC + standard metrics, vs standard metrics, by linear discriminant analysis AUC of CMD prevalence, including covariates in both models, after removal of variables with variance inflation factor > 5. CMD, cardiometabolic disease; AUC, area under the curve; PCs: principal components plus LV and RV global circumferential and longitudinal strains. Standard model: LV mass, RV ejection fraction, LV and RV global circumferential and longitudinal strains, and MAPSE and TAPSE. Covariates were: age at imaging, sex, height, weight, BMI and afterload (none removed).

| CMD | AUC | | DeLong test P-value |
| --- | --- | --- | --- |
|  | **PCs** | **Standard** |  |
| Atrial fibrillation (n=1088) | 0.77 | 0.75 | 2.8E-03 |
| Heart failure (n=341) | 0.85 | 0.83 | 8.2E-03 |
| Hypertrophic cardiomyopathy (n=26) | 0.84 | 0.83 | 0.85 |
| Dilated cardiomyopathy (n=32) | 0.94 | 0.92 | 0.27 |
| Ventricular arrhythmia (composite) (n=151) | 0.73 | 0.73 | 0.90 |
| Myocardial infarction or ischaemic heart disease (n=2389) | 0.77 | 0.77 | 0.84 |
| Diabetes mellitus (n=1656) | 0.82 | 0.80 | 3.4E-11 |
| Conduction disease (n=635) | 0.81 | 0.79 | 2.3E-04 |
