# Supplementary material for: Relationships between heart shape, function, and disease in 38,858 UK biobank participants
Source: J Cardiovasc Magn Reson. 2025 Jun 2;27(2):101919. doi: 10.1016/j.jocmr.2025.101919 (PMC12780292; doi:10.1016/j.jocmr.2025.101919)
Supplement: Supplementary file 3 — Supplementary material [file mmc3.pptx]

## Slide 1
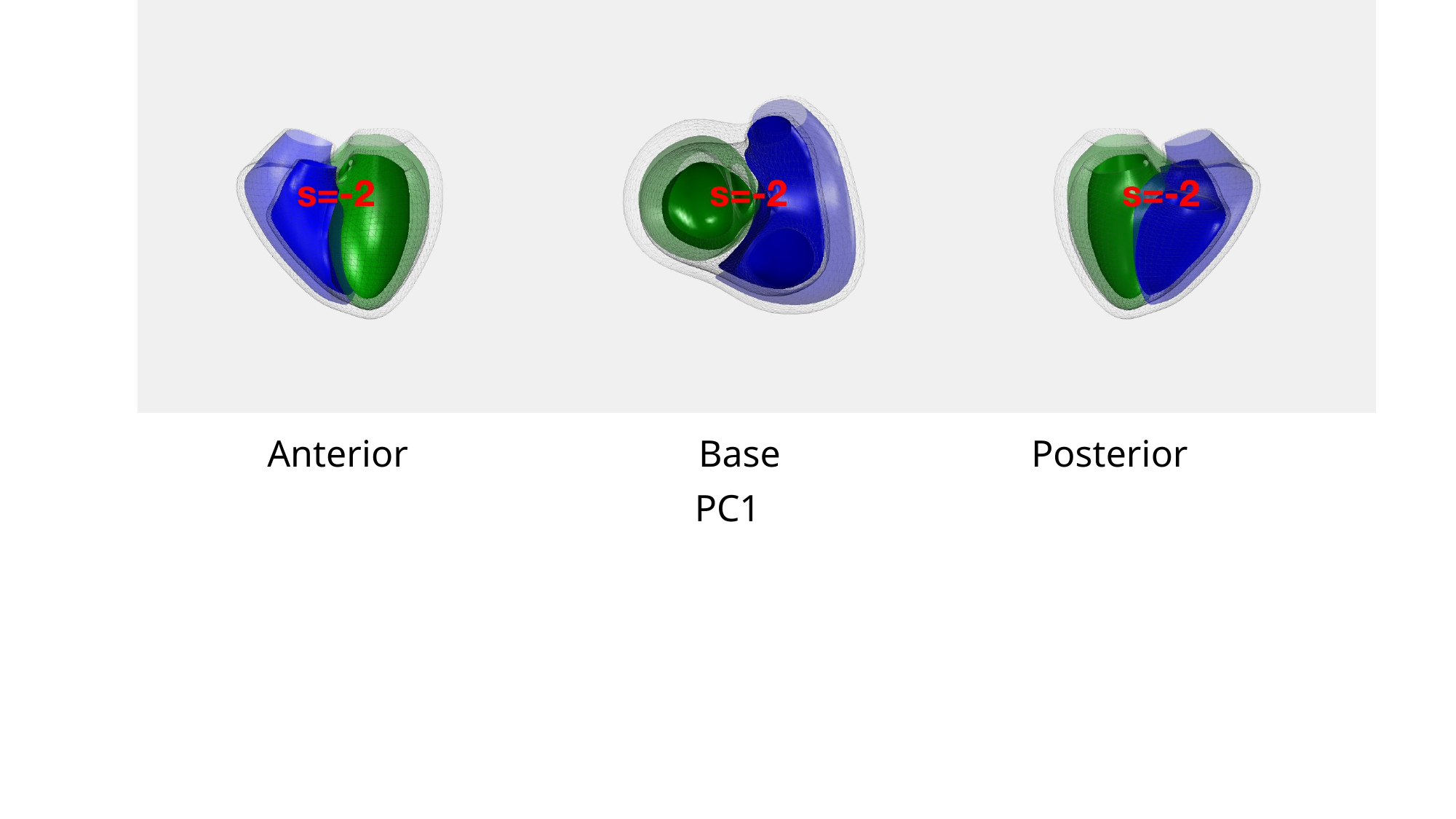

Anterior		 Base			Posterior
PC1

## Slide 2
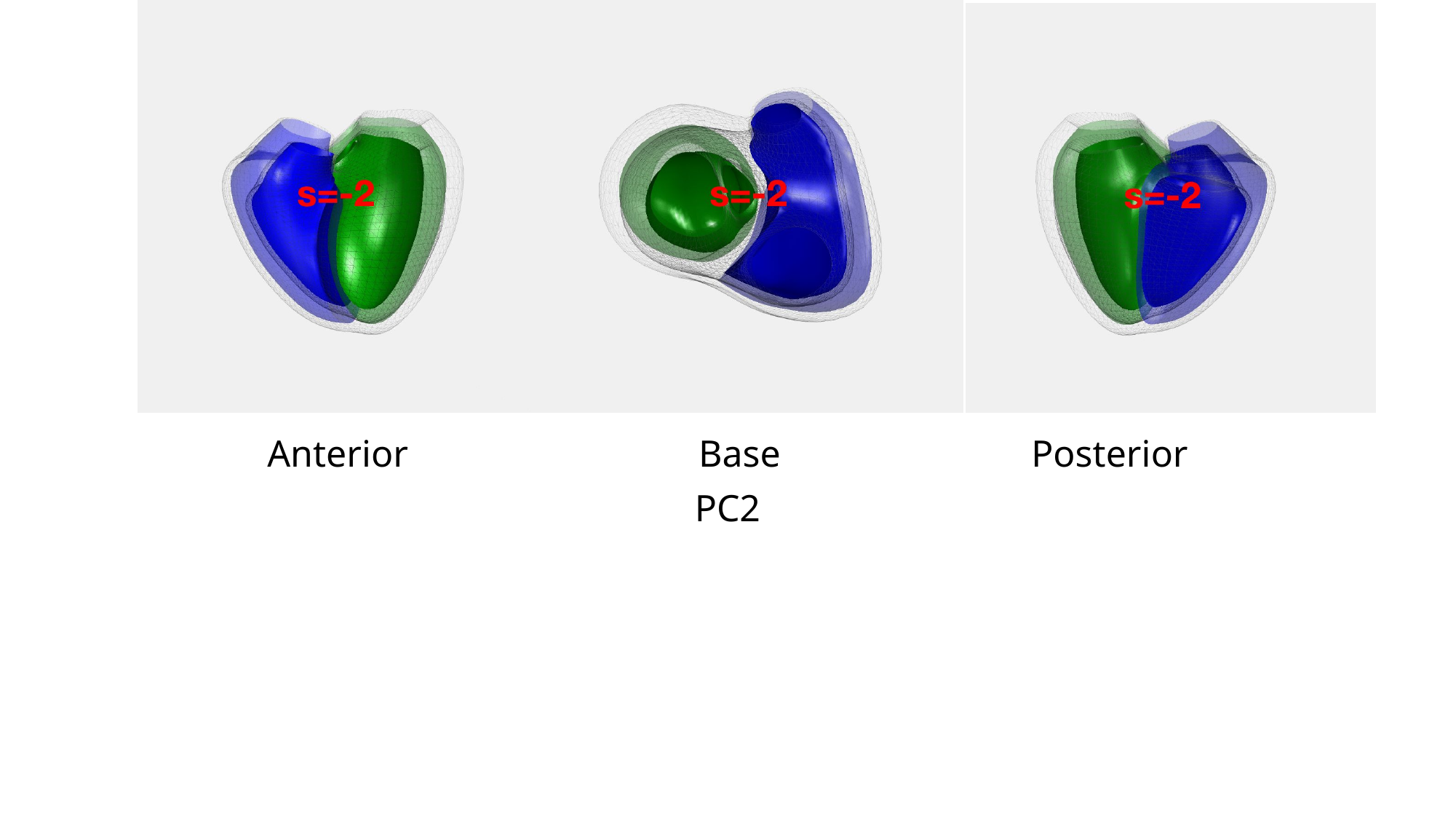

Anterior		 Base			Posterior
PC2

## Slide 3
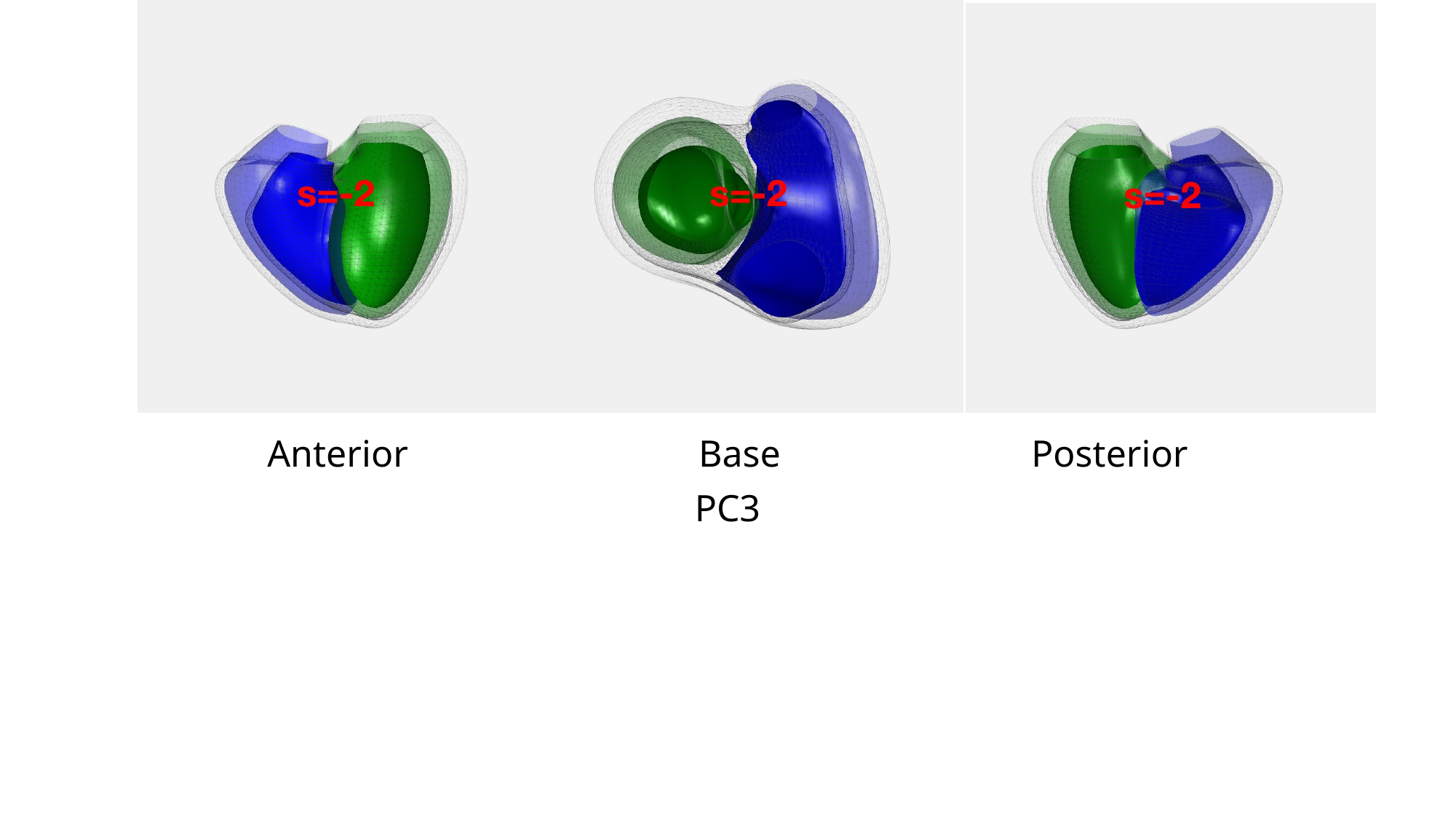

Anterior		 Base			Posterior
PC3

## Slide 4
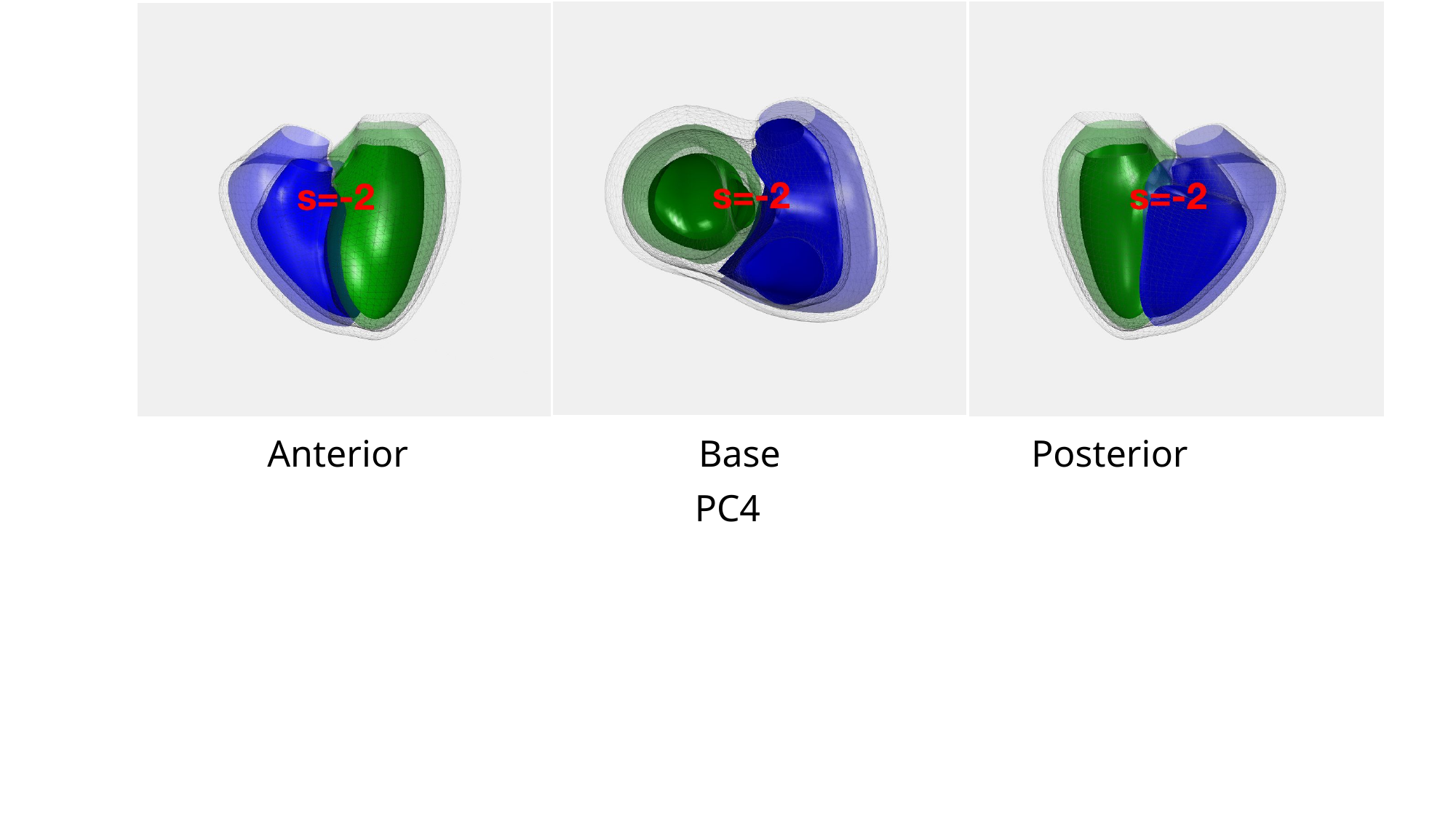

Anterior		 Base			Posterior
PC4

## Slide 5
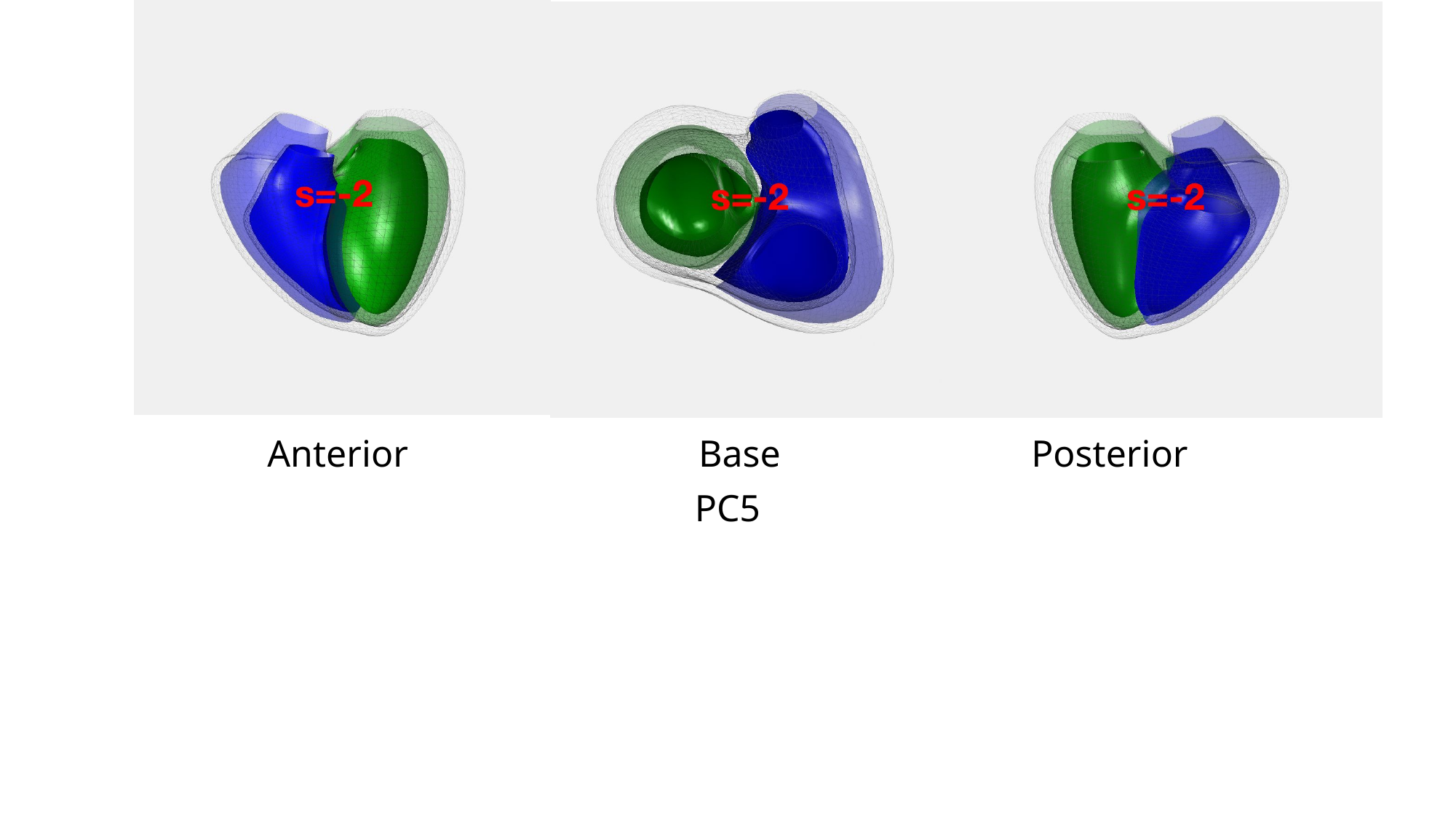

Anterior		 Base			Posterior
PC5

## Slide 6
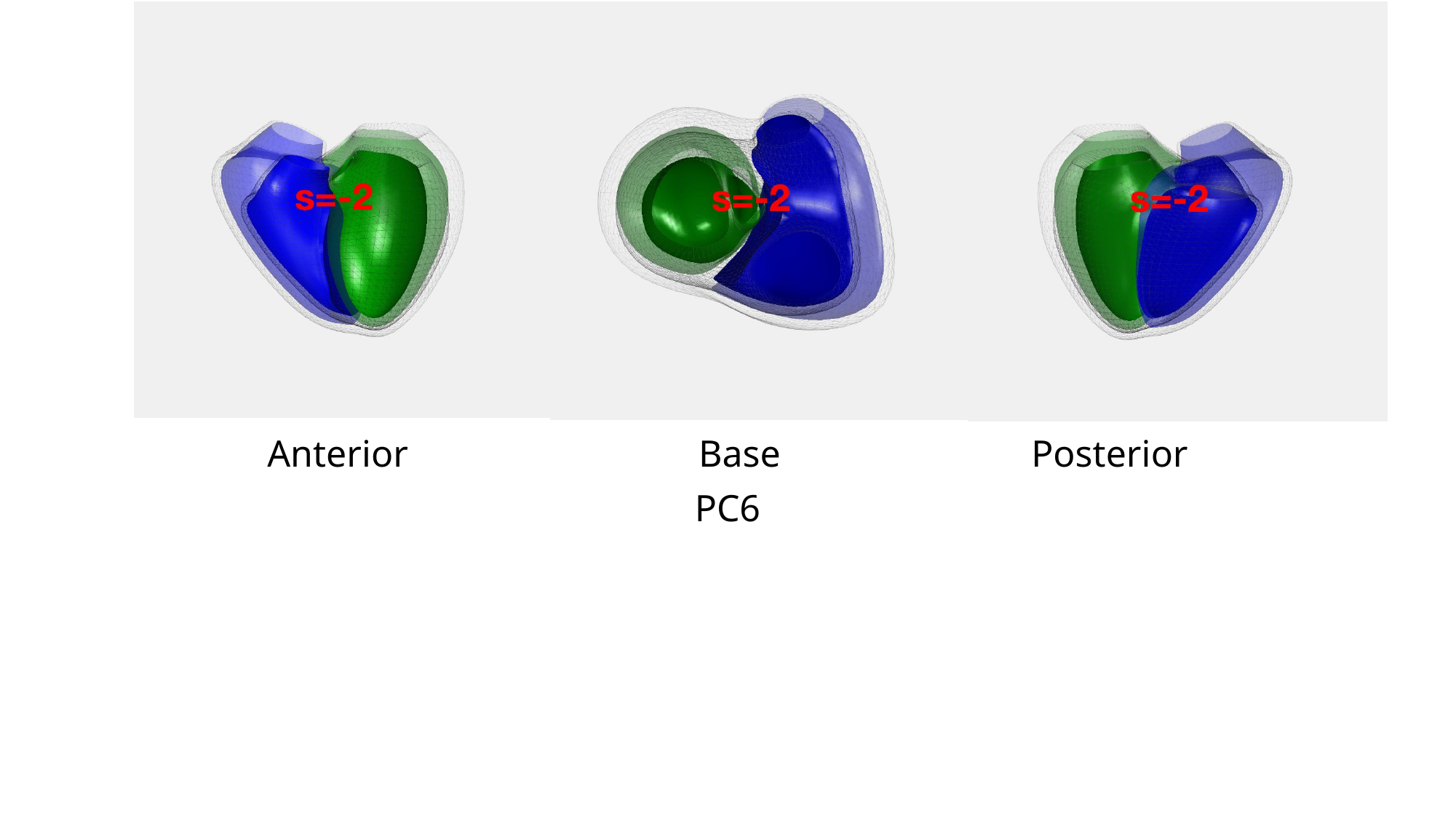

Anterior		 Base			Posterior
PC6

## Slide 7
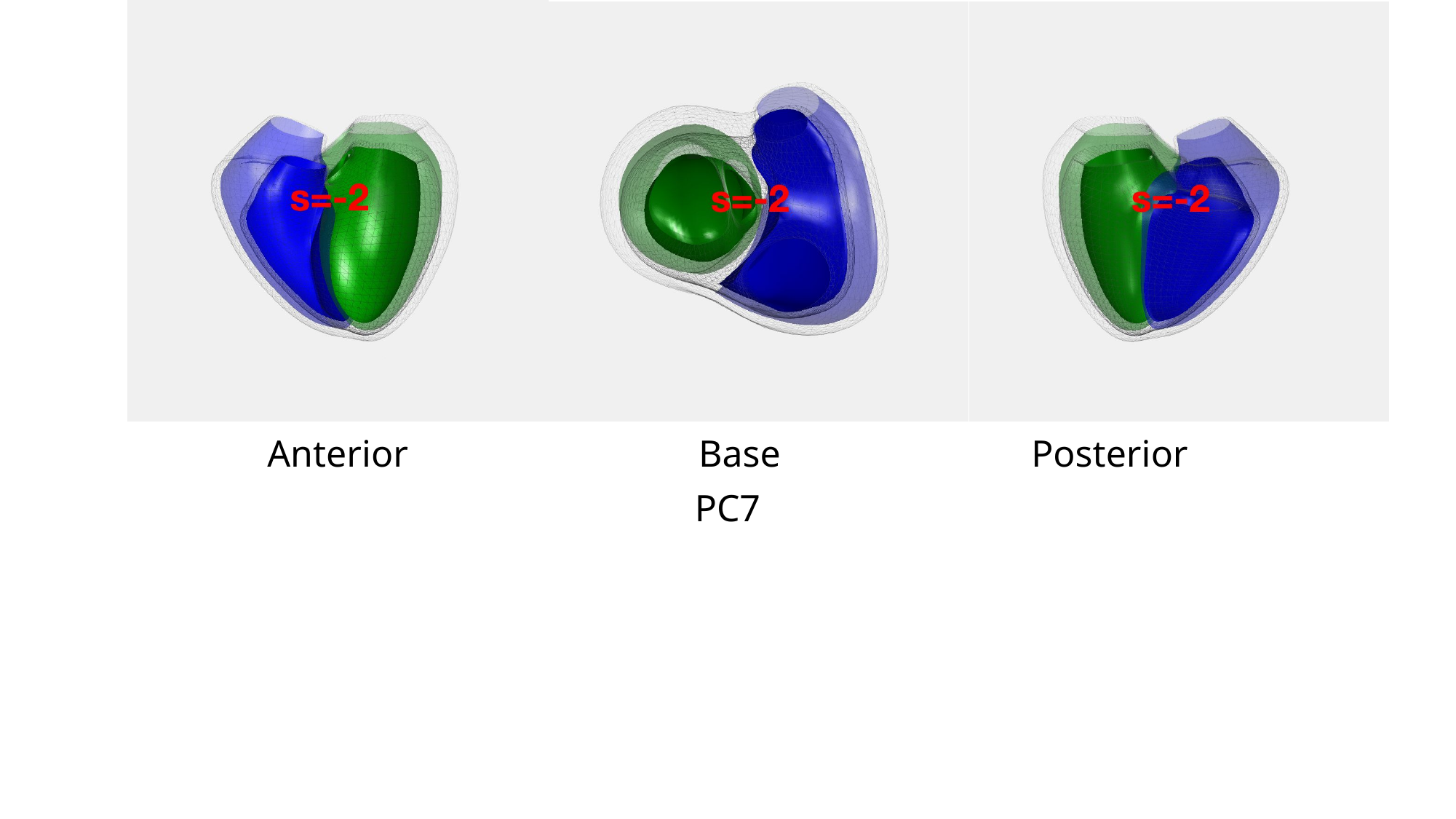

Anterior		 Base			Posterior
PC7

## Slide 8
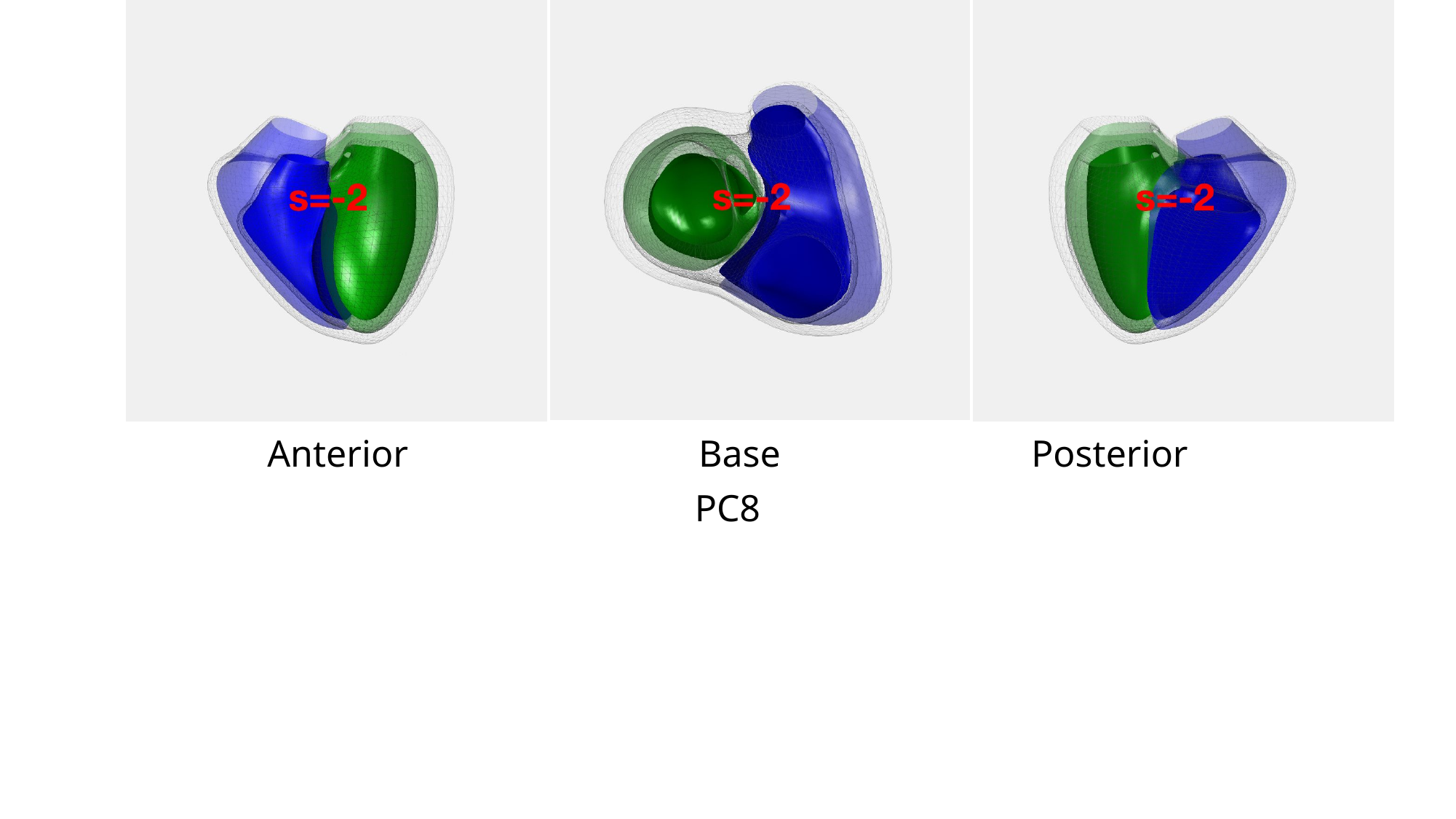

Anterior		 Base			Posterior
PC8

## Slide 9
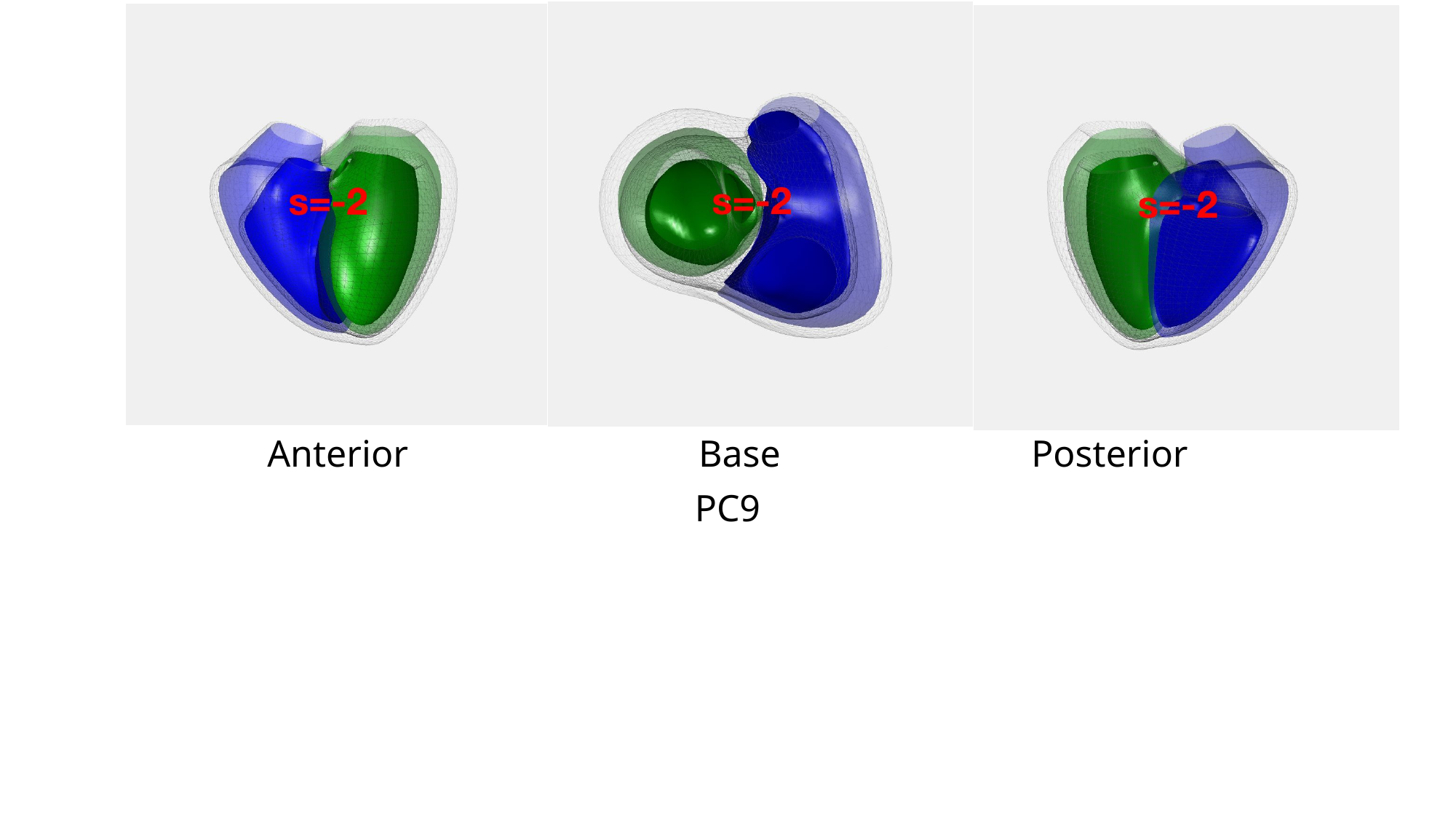

Anterior		 Base			Posterior
PC9

## Slide 10
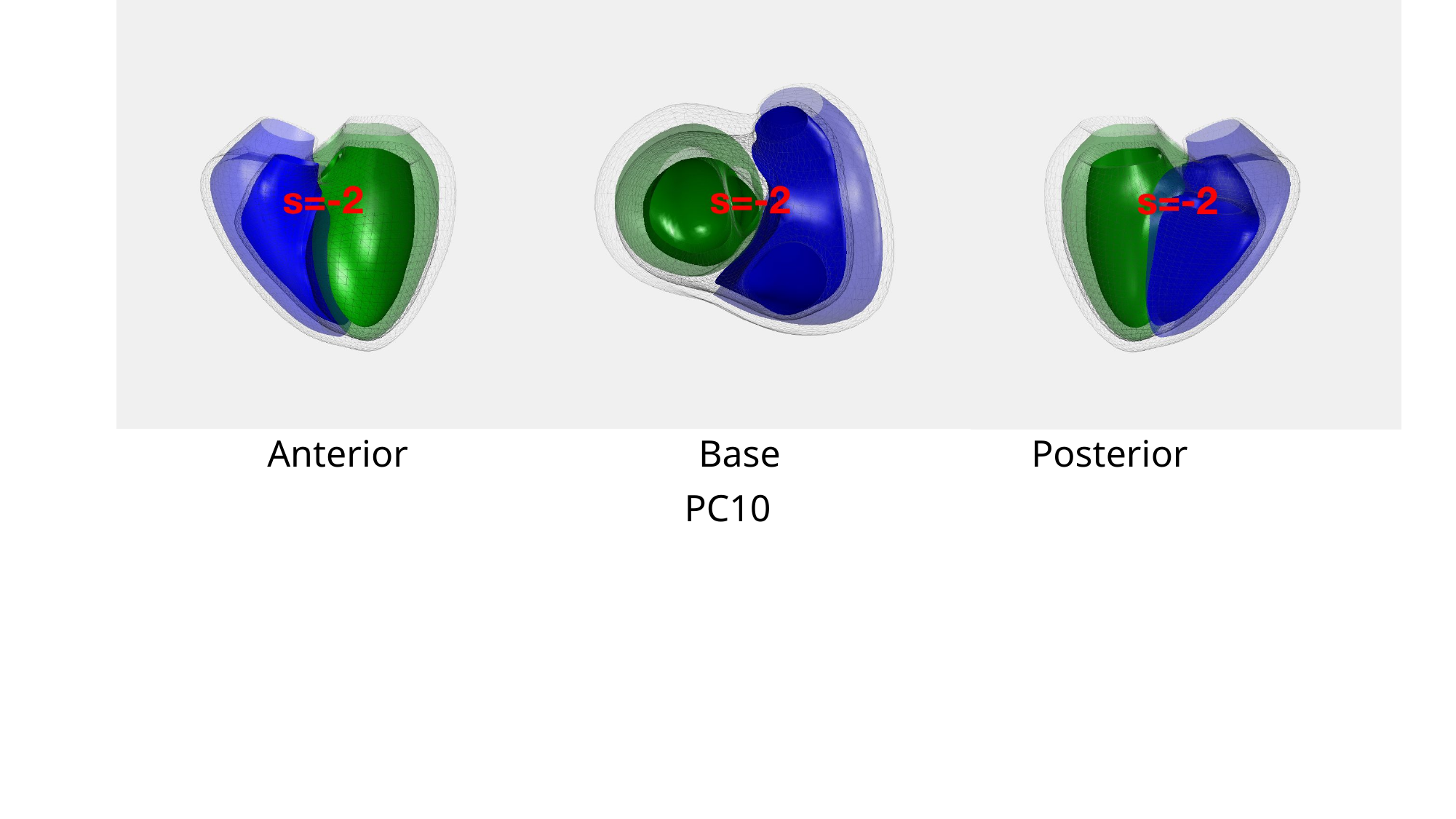

Anterior		 Base			Posterior
PC10

## Slide 11
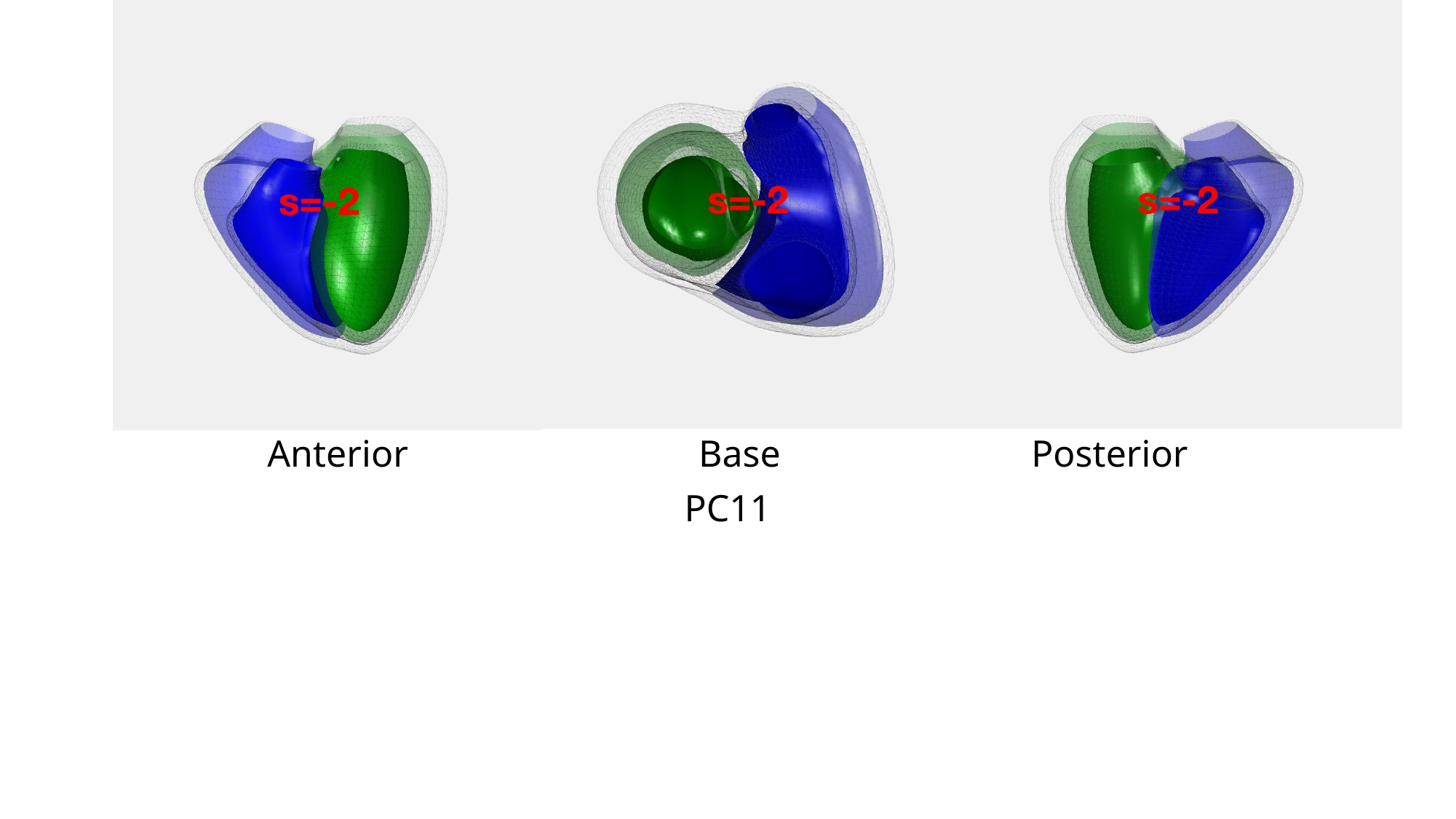

Anterior		 Base			Posterior
PC11

## Slide 12
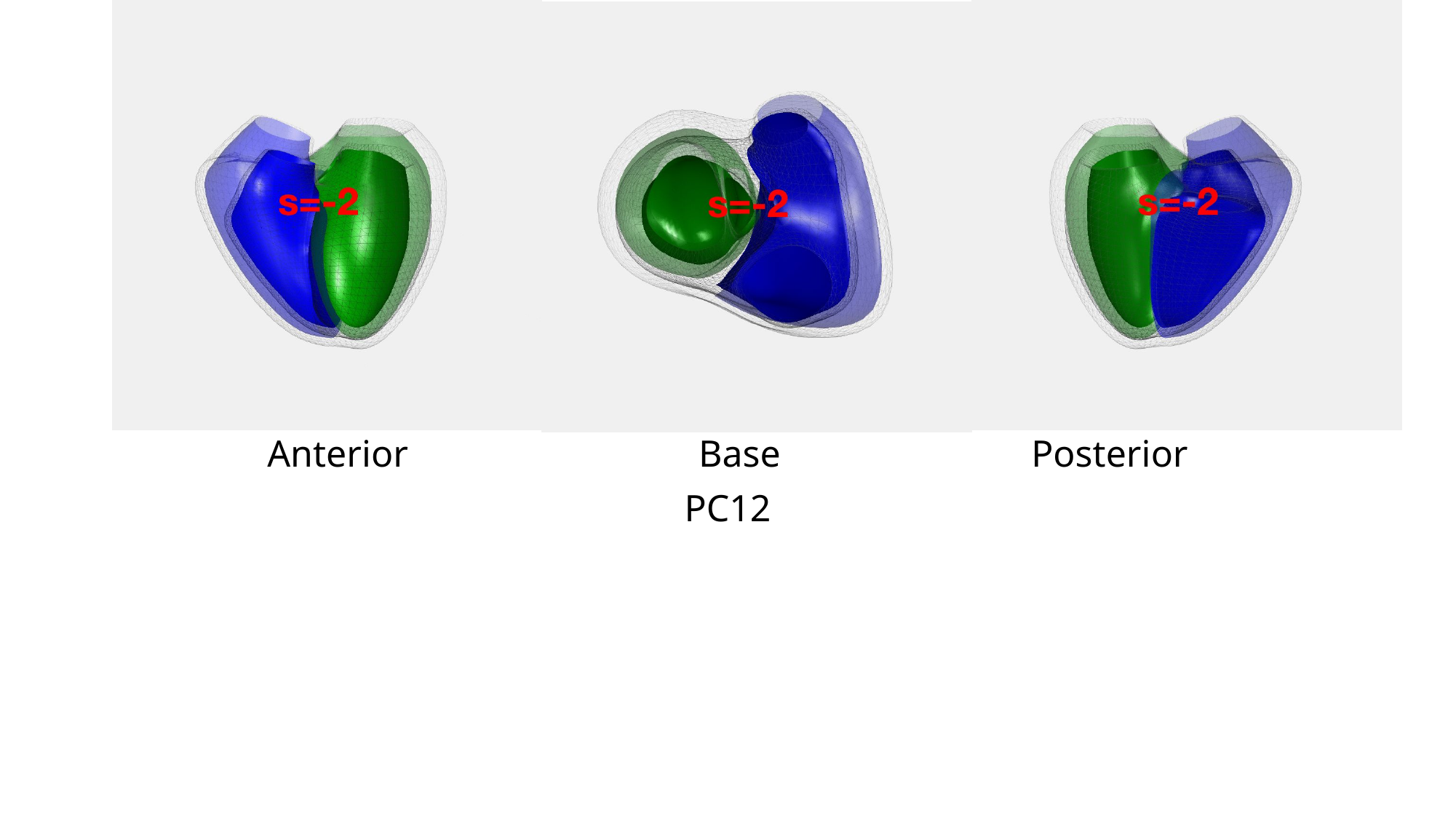

Anterior		 Base			Posterior
PC12

## Slide 13
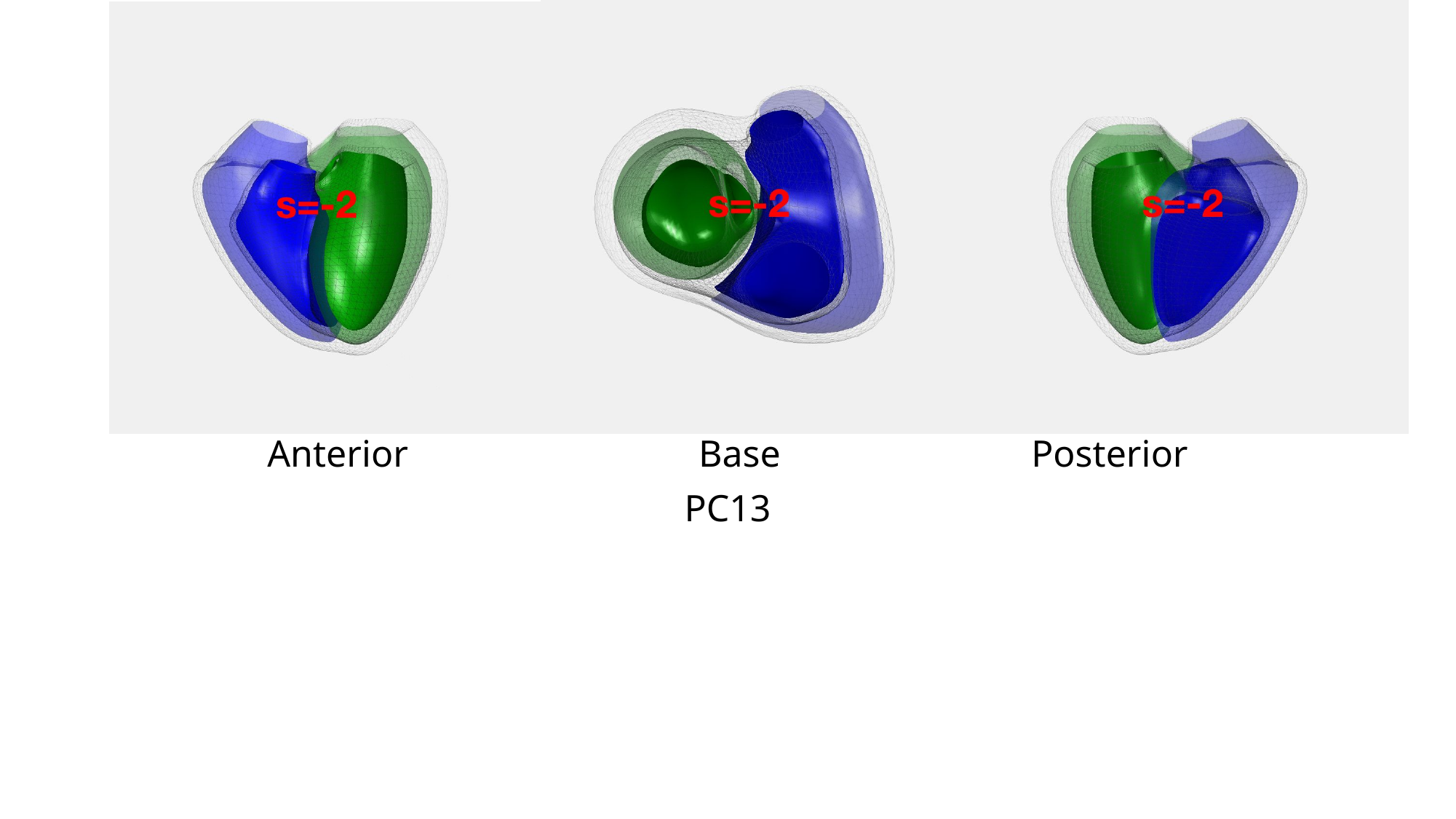

Anterior		 Base			Posterior
PC13

## Slide 14
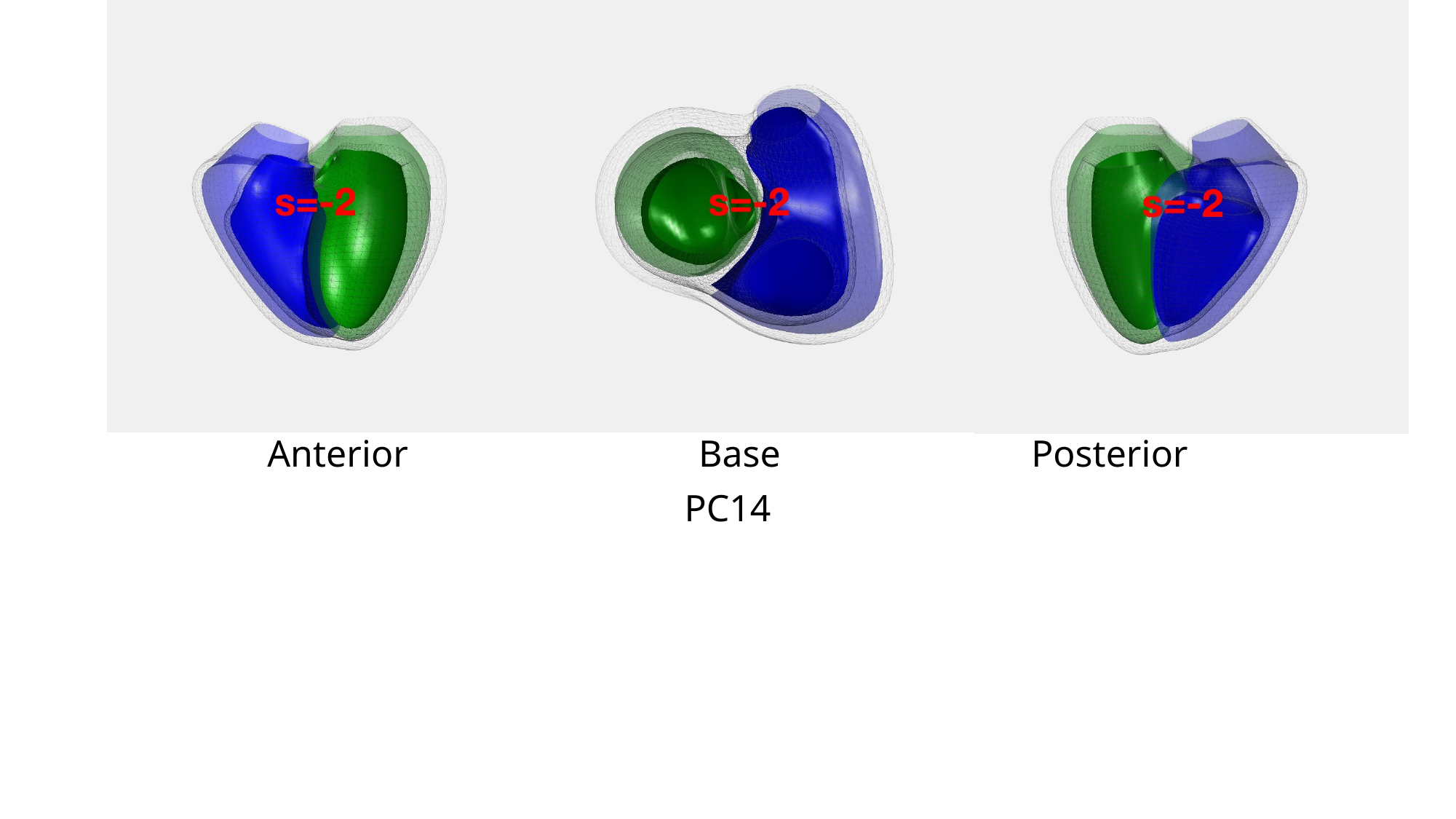

Anterior		 Base			Posterior
PC14

## Slide 15
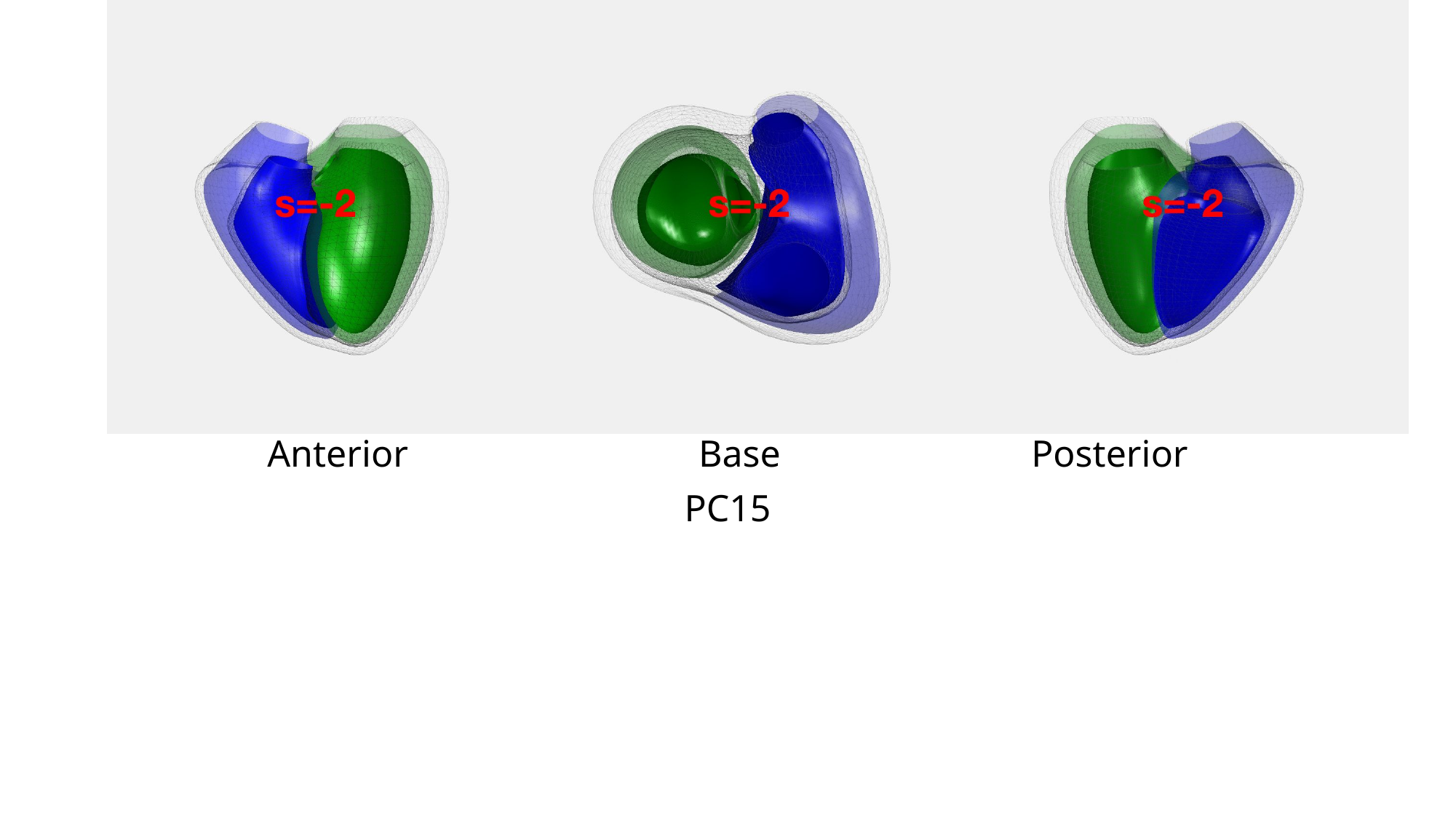

Anterior		 Base			Posterior
PC15

## Slide 16
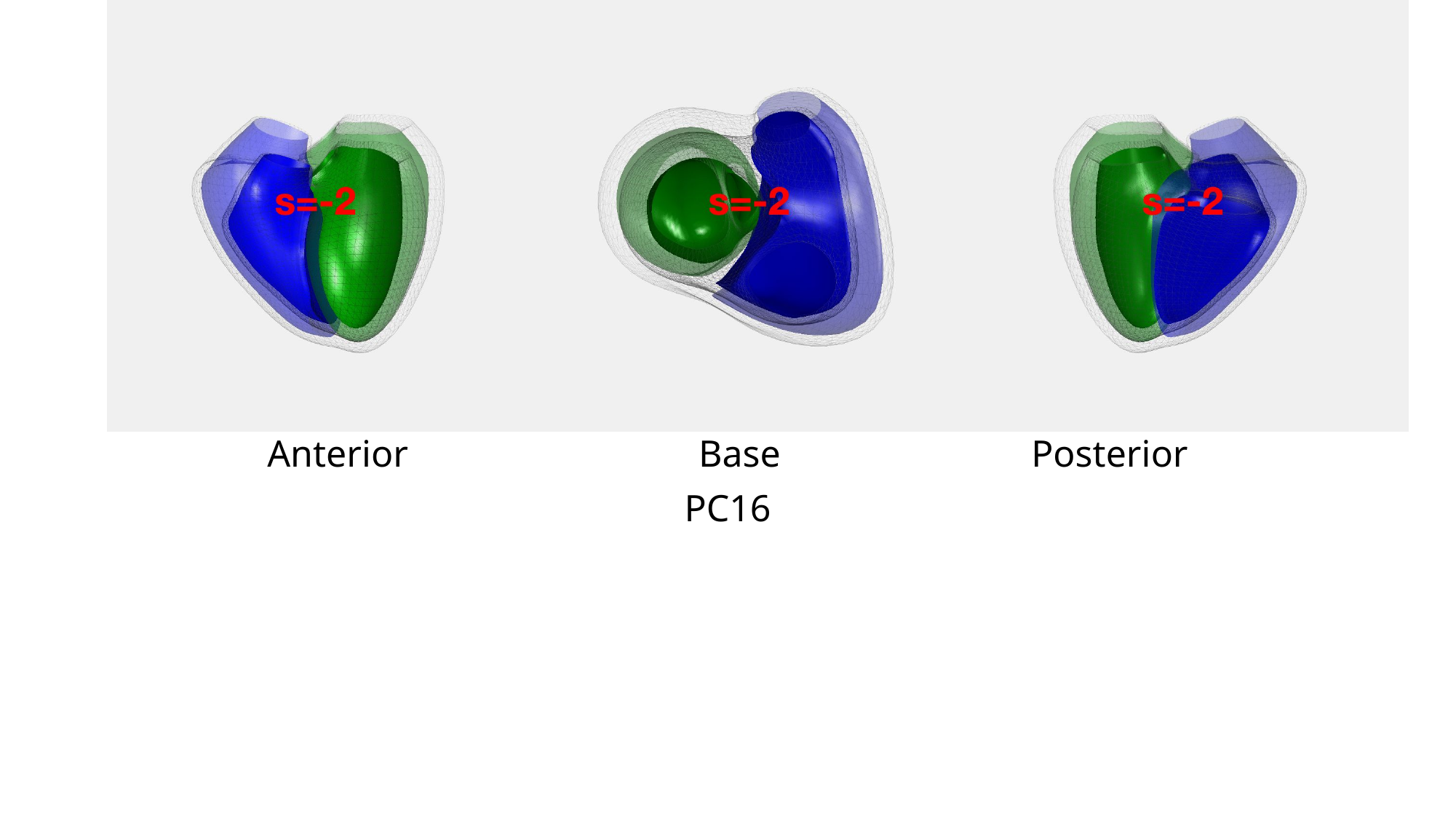

Anterior		 Base			Posterior
PC16

## Slide 17
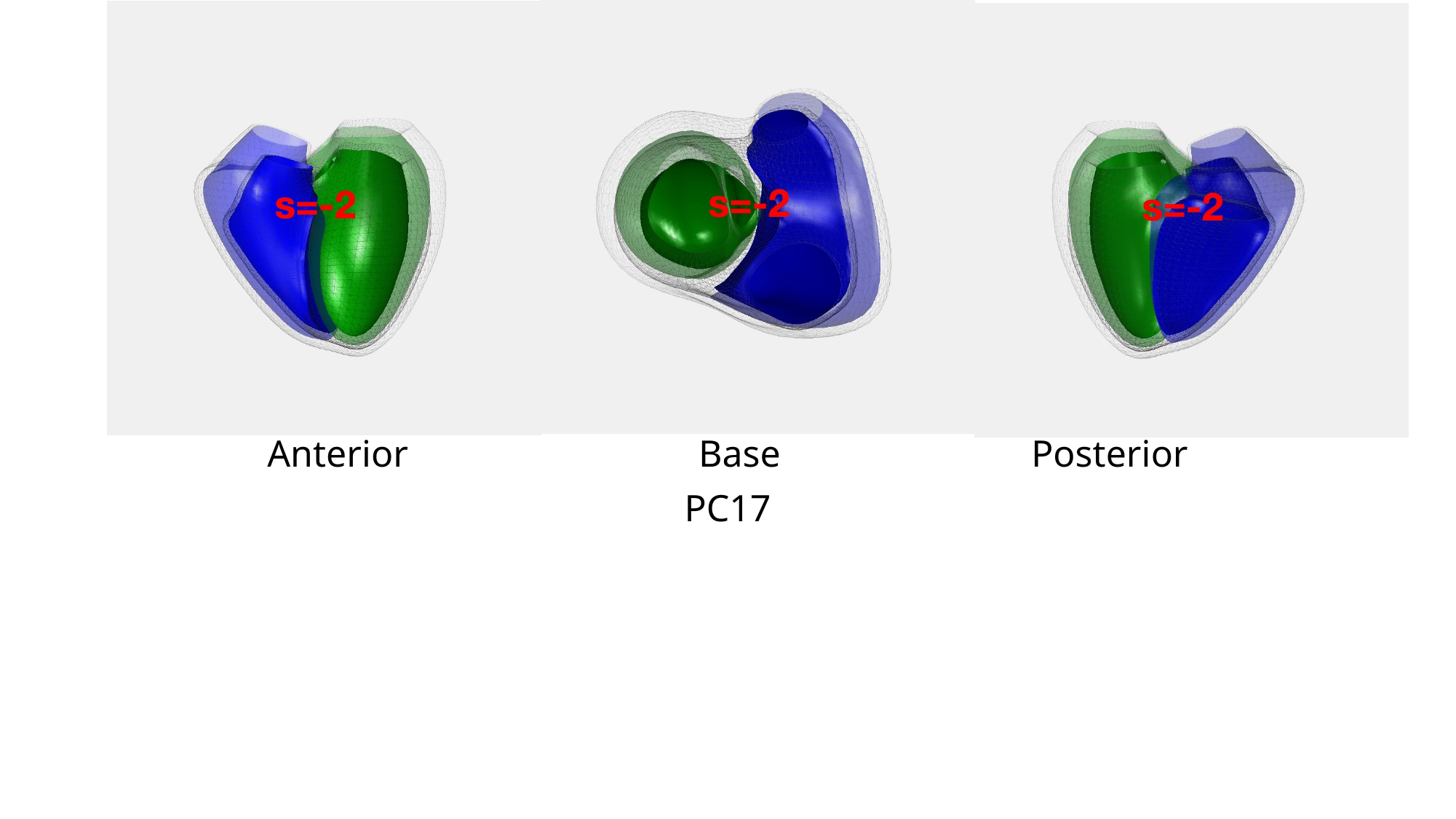

Anterior		 Base			Posterior
PC17

## Slide 18
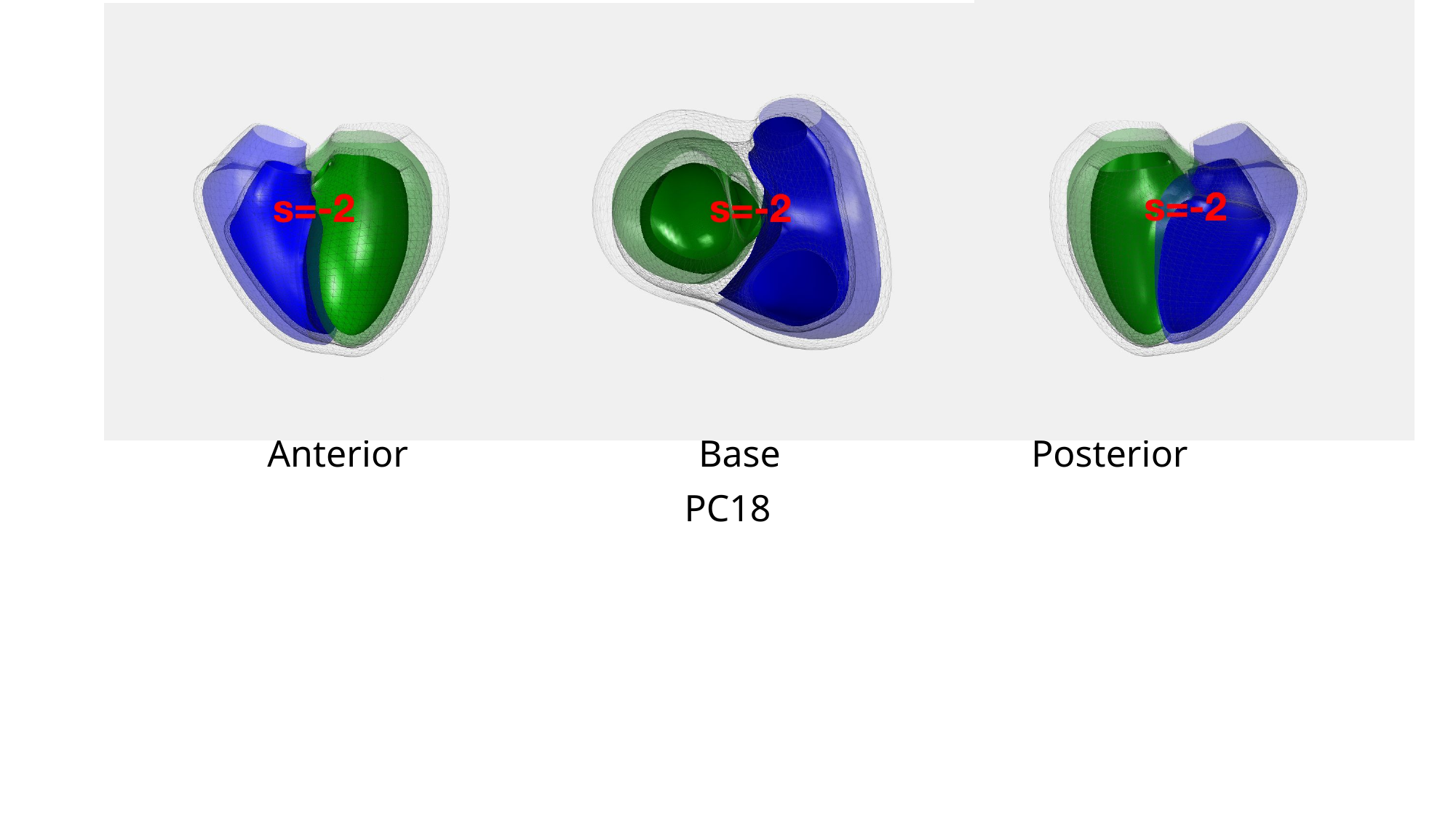

Anterior		 Base			Posterior
PC18

## Slide 19
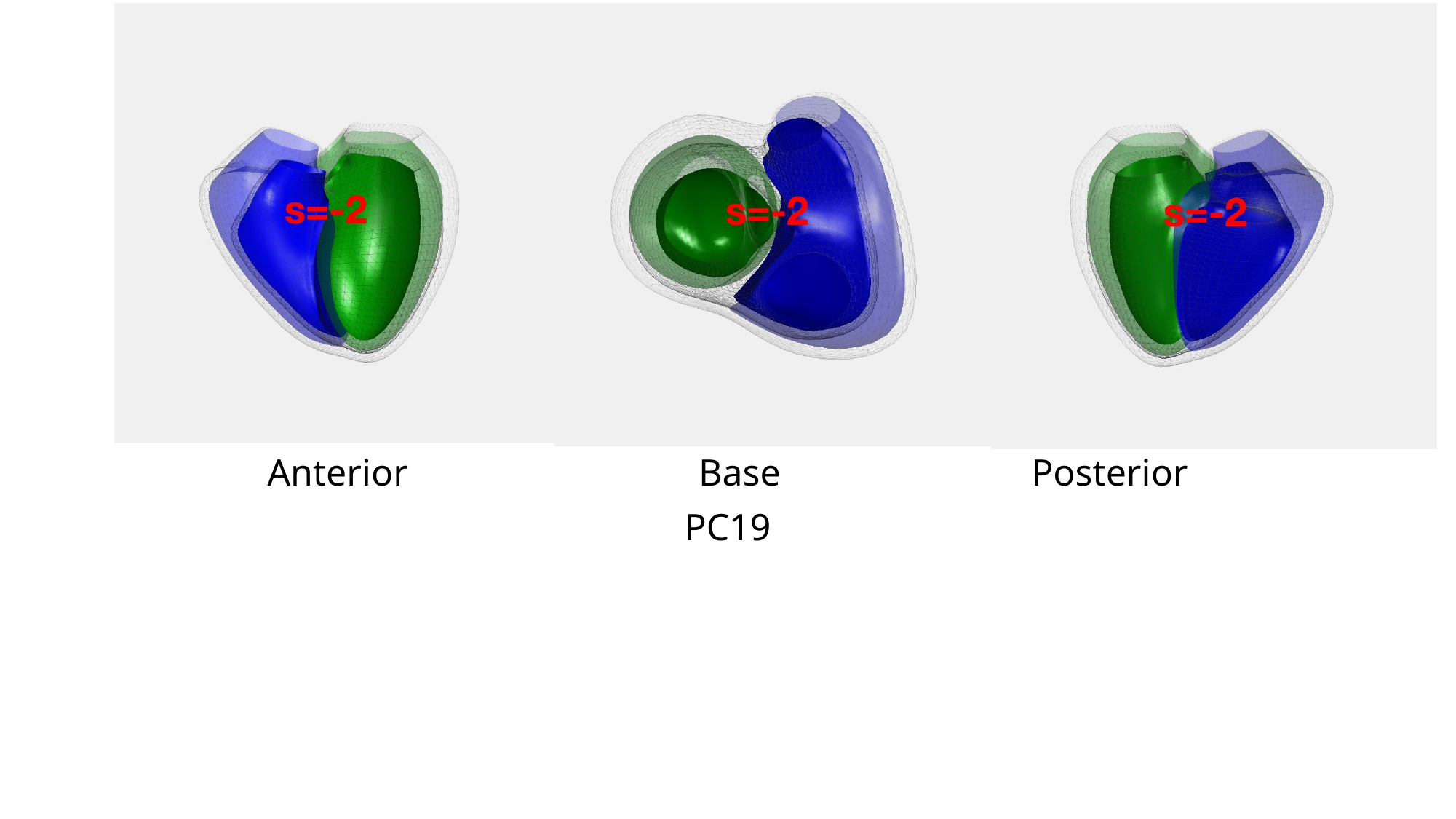

Anterior		 Base			Posterior
PC19

## Slide 20
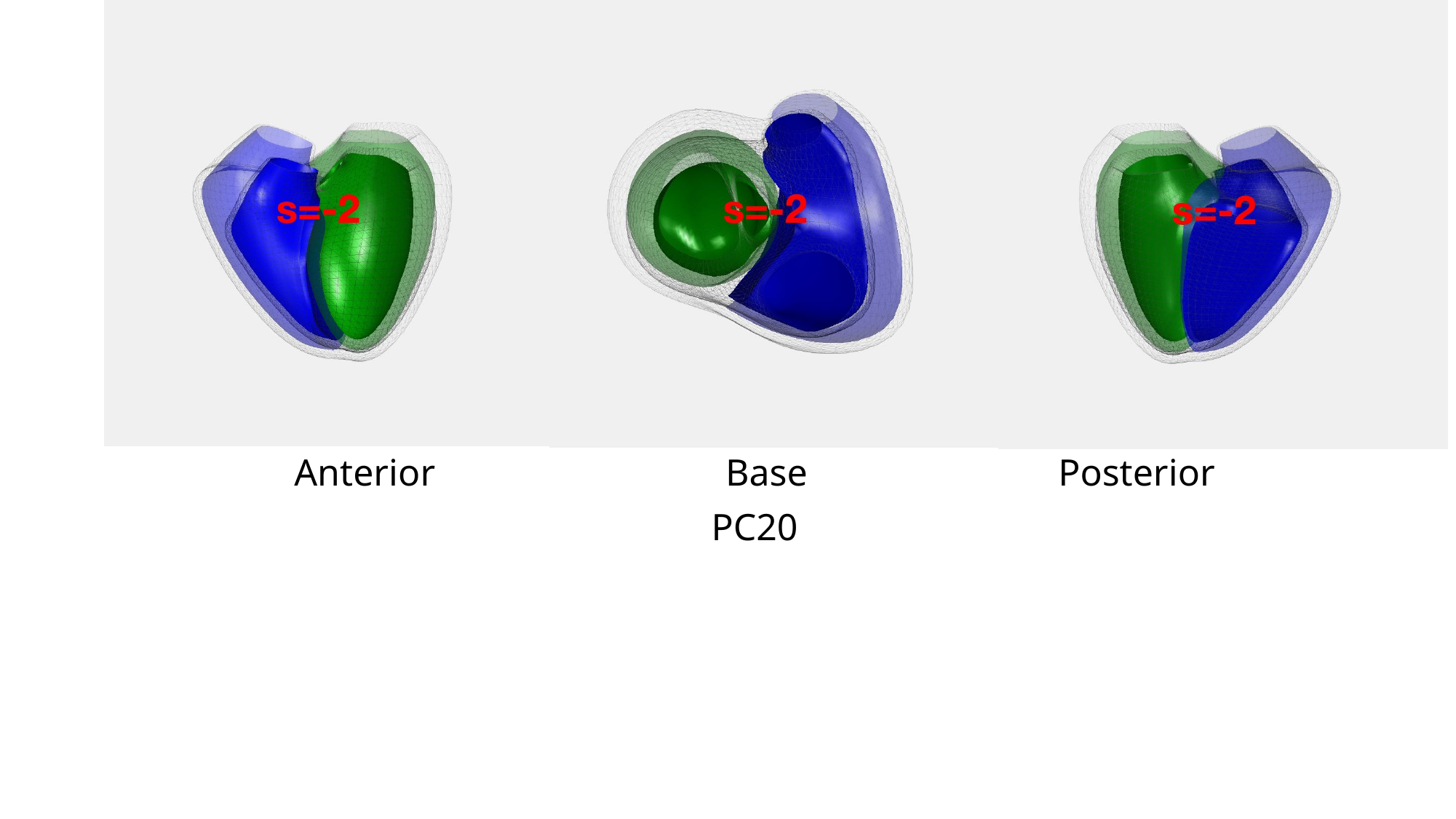

Anterior		 Base			Posterior
PC20

## Slide 21
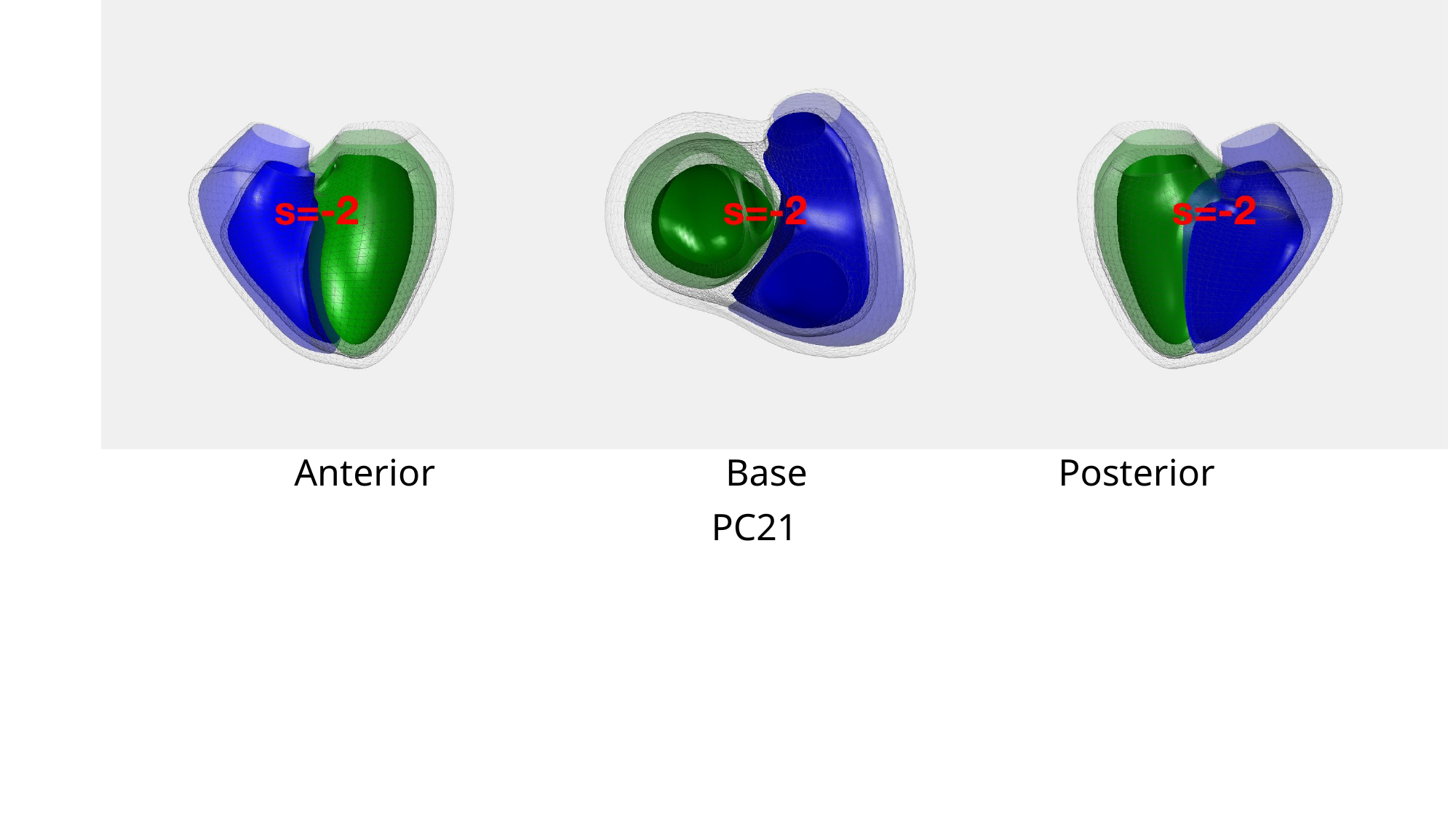

Anterior		 Base			Posterior
PC21

## Slide 22
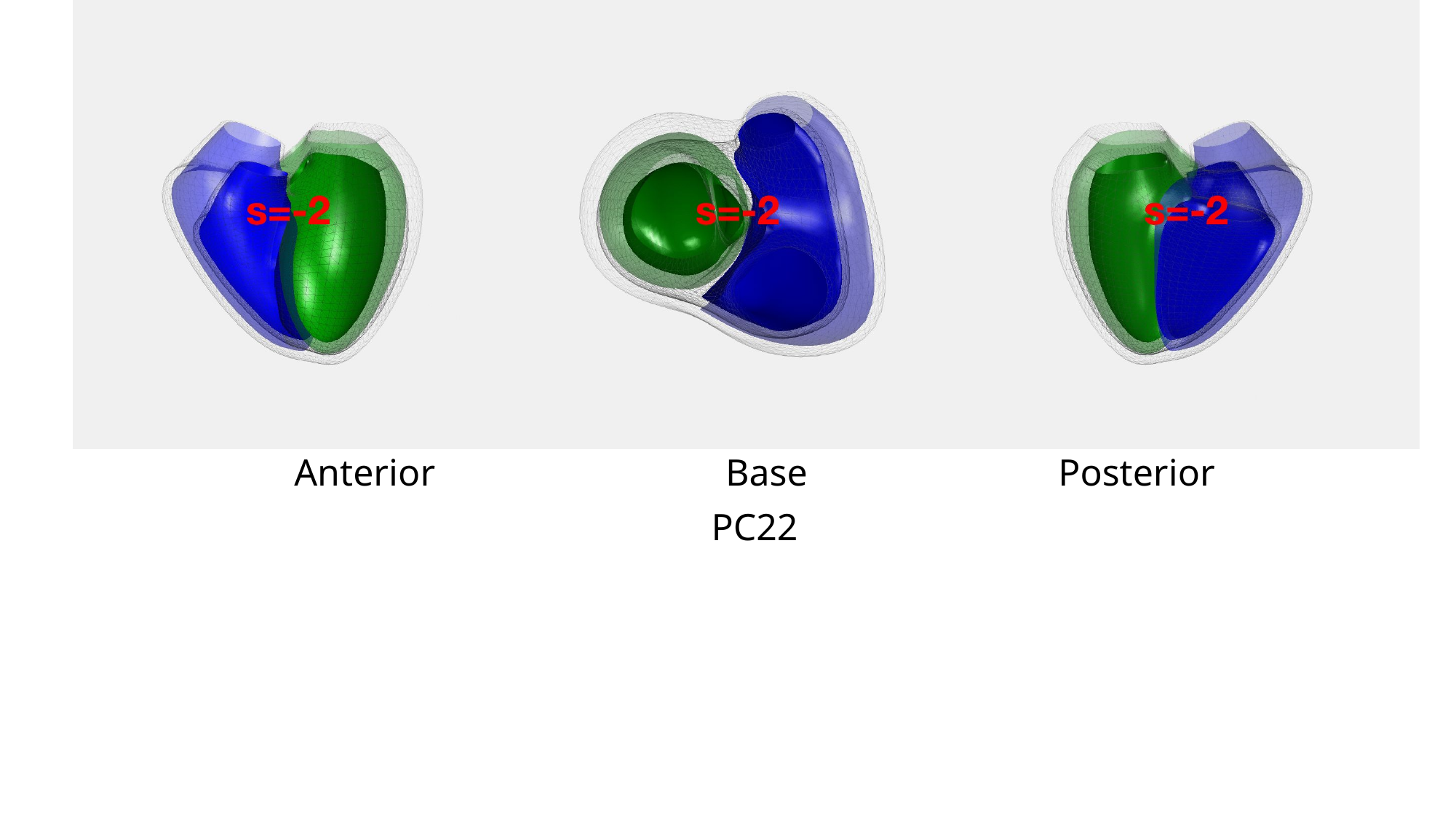

Anterior		 Base			Posterior
PC22

## Slide 23
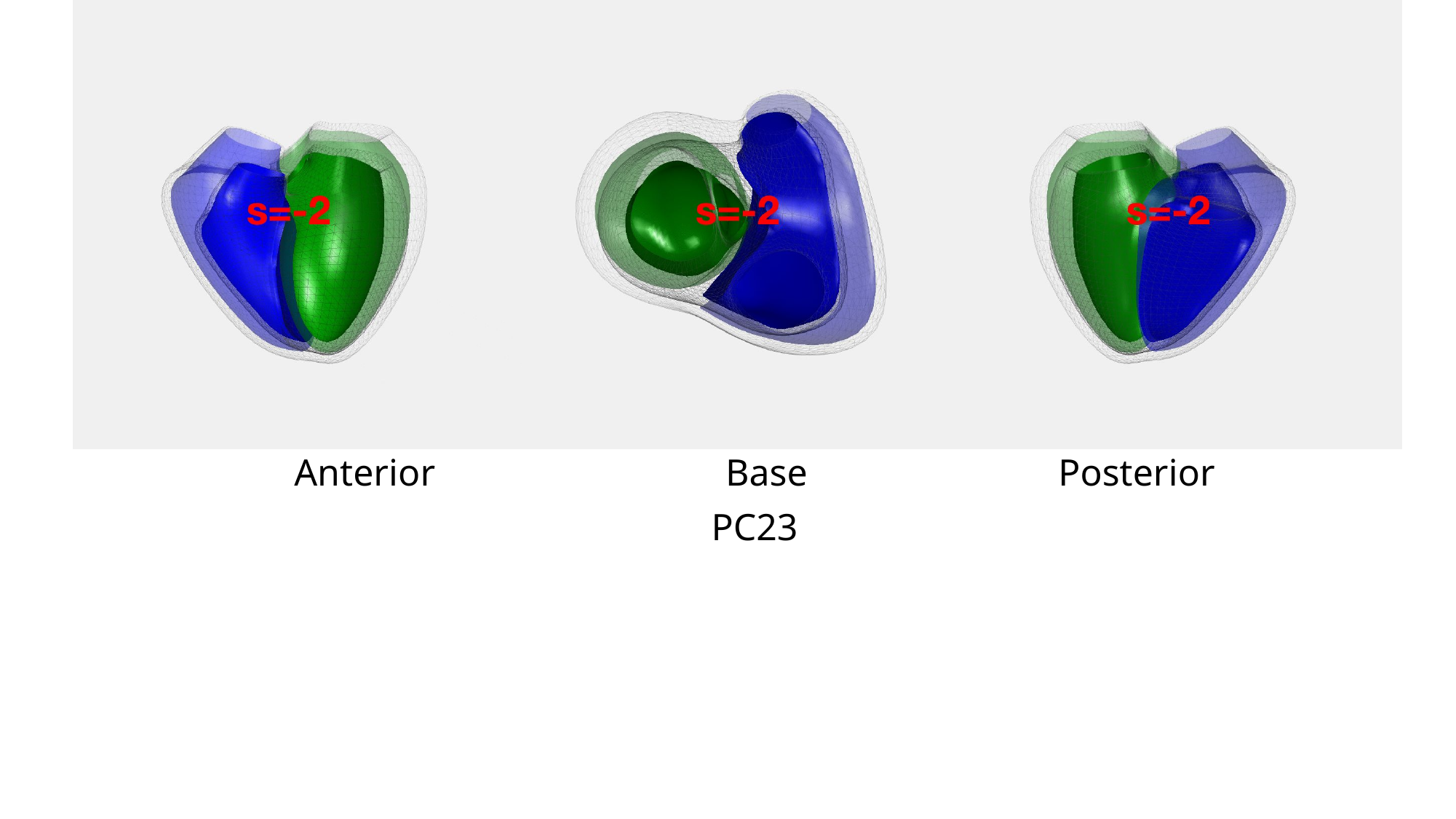

Anterior		 Base			Posterior
PC23

## Slide 24
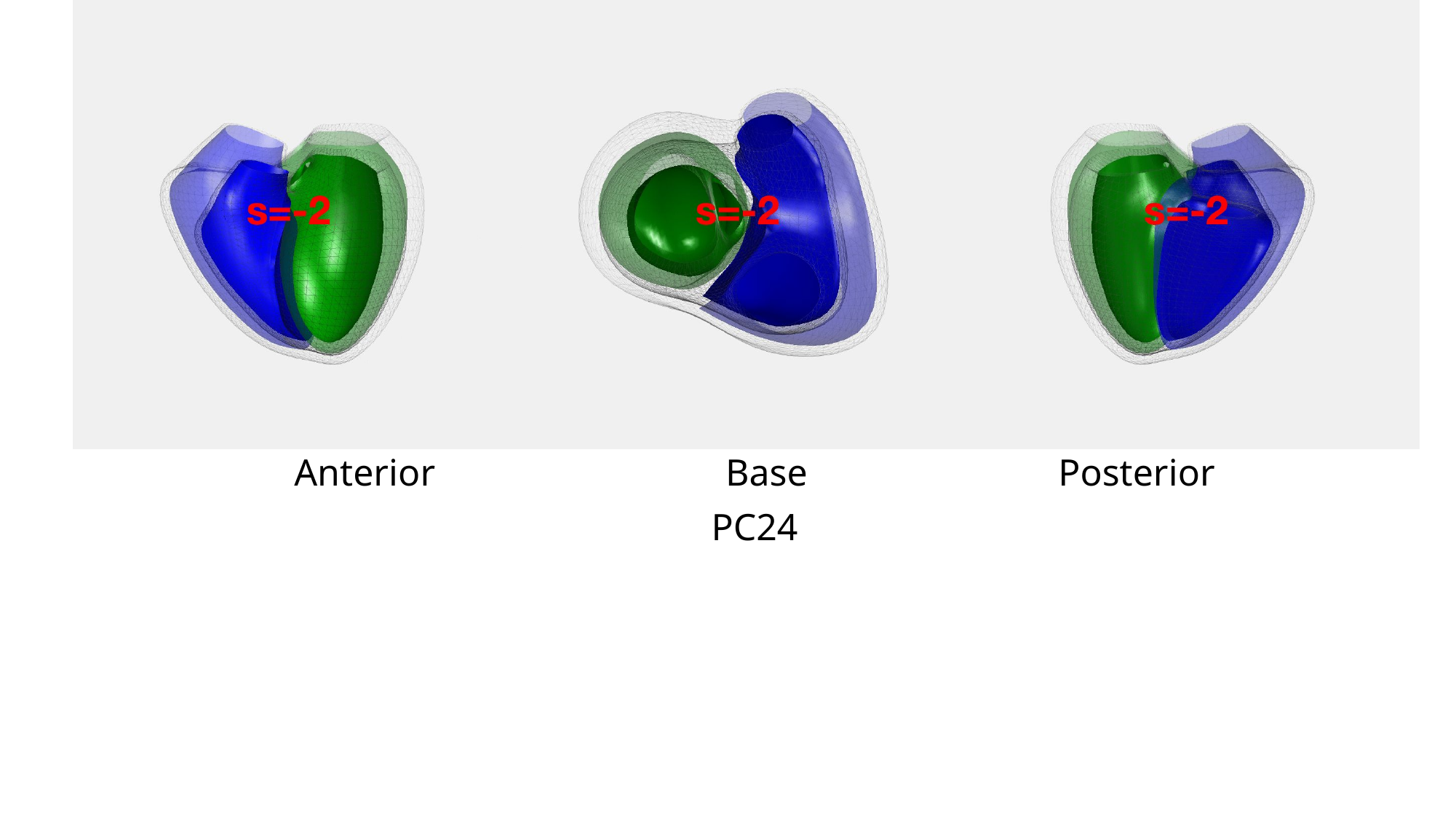

Anterior		 Base			Posterior
PC24

## Slide 25
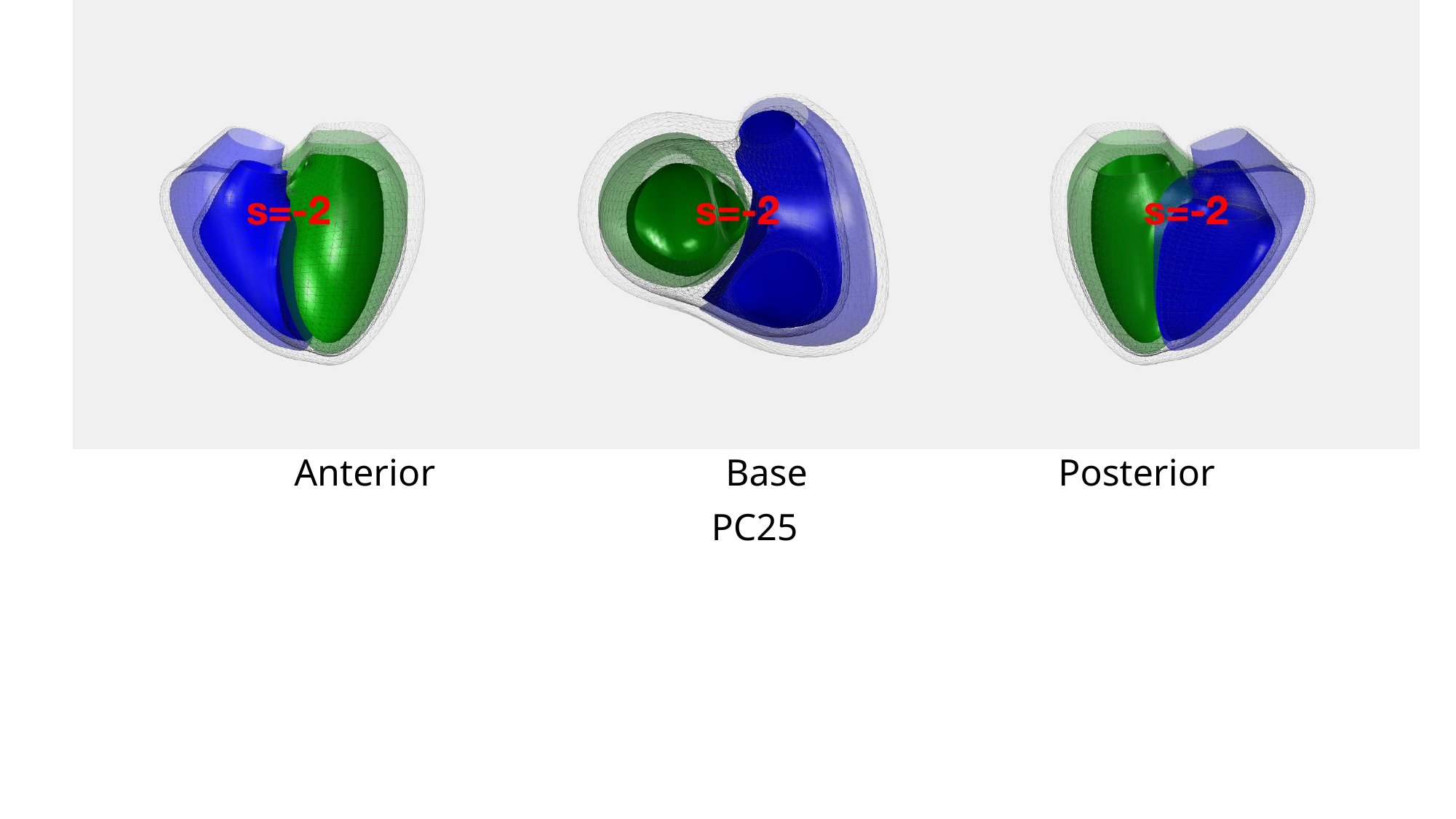

Anterior		 Base			Posterior
PC25
